# A Foundation Model for Sleep-Based Risk Stratification and Clinical Outcomes

**DOI:** 10.21203/rs.3.rs-6307069/v1

**Published:** 2025-04-10

**Authors:** Erhan Bilal, Matheus Lima Diniz Araujo, Kristen L. Beck, Catherine M. Heinzinger, Samer Ghosn, Carl Y. Saab, Nancy Foldvary Schaefer, Jeffrey L. Rogers, Reena Mehra

**Affiliations:** Digital Health, IBM Research, T.J. Watson Research Center, Yorktown Heights, USA; Sleep Disorders Center, Neurological Institute Cleveland Clinic Foundation, Cleveland, USA; Digital Health, IBM Research, IBM Research Almaden Lab, San Jose, USA; Sleep Disorders Center, Neurological Institute Cleveland Clinic Foundation, Cleveland, USA; Department of Biomedical Engineering, Cleveland Clinic Foundation, Cleveland, USA; Department of Biomedical Engineering, Cleveland Clinic Foundation, Cleveland, USA; School of Medicine, Case Western Reserve University, Cleveland, USA; Department of Engineering, Brown University, Providence, USA; Sleep Disorders & Epilepsy Centers, Neurological Institute Cleveland Clinic Foundation, Cleveland, USA; Digital Health, IBM Research, T.J. Watson Research Center, Yorktown Heights, USA; Division of Pulmonary, Critical Care, and Sleep Medicine, University of Washington, Seattle, USA

## Abstract

Clinical diagnosis of sleep disorders, which are recognized contributors to morbidity and mortality, often relies on polysomnography (PSG) data. However, the vast physiologic data collected during PSG is underutilized, presenting a key opportunity to enhance characterization of sleep dysfunction and predict clinical outcomes. We introduce a sleep foundation model that uniquely integrates PSG time-series signals and electronic medical record data. Using a diverse dataset (n=10,000; mean observation period 14.5±7.1 years), our transformer-based model generates data-driven representations of latent physiological patterns. When clustered, we identified subpopulations with differential health trajectories. The highest risk-group exhibited strong correlations with all-cause mortality (unadjusted hazard ratio [HR] 4.83, 95% confidence interval [CI] 3.60–6.50, p<0.001) as well as cardiovascular outcomes and neurological outcomes, even after accounting for traditional measures. External validation in a National Sleep Research Resource cohort confirmed findings. We created a novel, clinically applicable framework leveraging information-dense PSG data to inform risk stratification and predict health outcomes beyond traditional methods.

## INTRODUCTION

Sleep is fundamental to health and well-being. Conversely, sleep disorders— associated with a range of physical, psychiatric, and cognitive symptoms— increase the risk of cardiovascular, metabolic, respiratory, and neurological conditions, as well as mortality. Reliable and accurate assessment of sleep and its associated clinical outcomes in a comprehensive, data-driven manner is a challenge, mainly due to the number of different disorders and their variable effects on general health, comorbidities, and genetic predisposition of an individual.

Diagnosing sleep disorders requires a comprehensive sleep and medical history supplemented by validated, self-assessment instruments and sleep diary, physical examination, and, in many cases— such as sleep disordered breathing (SDB) or central disorders of hypersomnolence— a diagnostic sleep study. A laboratory-based overnight sleep study, known as a polysomnogram (PSG), is conducted by a trained technologist and involves direct, real-time, continuous physiologic monitoring of electroencephalography (EEG), electrooculography (EOG), chin and limb electromyography (EMG), electrocardiography (EKG), oxygen saturation, oral and nasal airflow, respiratory effort (inductance plethysmography), position, snoring, video, and, in some cases, carbon dioxide monitoring. These rich and complex multimodal physiological parameters are collected over the sleep period which typically range from six to eight hours and are later interpreted by a physician. Yet current diagnostic criterion for SDB, as an example, is based solely on the apnea-hypopnea index (AHI)^1^, an essentially unidimensional measure of the frequency of upper airway closure. Hence, the richness and complexity of sleep physiological data are not efficiently and systematically incorporated into the ascertainment of clinical diagnoses or decision-making.

Several clinic-based and epidemiologic studies have examined the utility of PSG-based clustering of aggregate indices such as the AHI, arousal index, and sleep stages scored and annotated by technologists to serve as predictors of clinical outcomes^2–4^. The fidelity of this approach, however, is limited due to its reliance on coarse aggregate PSG measures, which depend on individual technologist interpretations rather than a deeper, more direct approach that leverages the information contained in the continuous multimodal physiologic dataset. Furthermore, in other areas of medicine, machine learning (ML), particularly clustering techniques, has revealed hidden disease subtypes, improving risk stratification and personalized treatment. For example, unsupervised learning has identified distinct metabolic subtypes of diabetes, refining treatment beyond the standard Type 1 and Type 2 classifications^5^. Similarly, clustering techniques applied to neuroimaging and genomic data have uncovered Alzheimer’s disease subgroups with varying disease trajectories and treatment responses^6^. However, traditional ML models often rely on predefined feature selection and task-specific architectures, limiting their ability to generalize across populations and adapt to new data.

To address these limitations, we introduce a foundation model for sleep health, designed to uncover latent physiological patterns associated with long-term disease development. Unlike conventional approaches, foundation models process large-scale, high-dimensional data without requiring extensive manual feature engineering, making them more scalable and generalizable. In this study, we leveraged 10,000 high-resolution PSG studies conducted at the Cleveland Clinic Sleep Disorders Center, paired with detailed longitudinal electronic medical records (EMR) spanning up to a decade, to train a transformer-based model that generates high-dimensional representations (i.e. embeddings) of a full night of sleep^7^. The model was trained specifically to ensure that these representations capture the intricate relationships between sleep architecture, respiratory events, and key physiological parameters.

Using clustering techniques, we identified distinct risk groups with significant differential longitudinal health trajectories. To validate our approach used in this clinical cohort, we examined the highest identified risk group in the multicenter, geographically diverse NIH/NHLBI Sleep Heart Health Study (SHHS)^8^ population-based cohort from the National Sleep Research Resource,^9^ confirming its strong association with adverse clinical outcomes. Our results demonstrate that learned sleep embeddings can effectively stratify patients into distinct risk groups, each characterized by unique disease-specific signatures that predict poor health outcomes, including cardiovascular disease, neurological disorders, and all-cause mortality. This work established a scalable, automated framework for labeling and analyzing PSG, introducing an independent risk classification schema that advances personalized sleep medicine beyond conventional methods.

## RESULTS

### Approach Overview

In this work, we sought to develop an automated time-series approach leveraging raw PSG data to generate more comprehensive patient characterizations than traditional, summary-based measures (e.g., AHI). Specifically, our study follows a three-step process: representation learning, patient stratification, and outcome analysis. First, we train a transformer-based foundation model on raw PSG time-series data, incorporating technologist annotations for sleep staging and respiratory events. This model learns high-dimensional representations (i.e. embeddings) that capture the intricate physiological patterns underlying sleep.

Next, we use these embeddings to identify distinct patient subgroups through unsupervised clustering, revealing patterns that are not apparent from conventional PSG metrics. Finally, we evaluate the clinical relevance of these subgroups by analyzing their longitudinal health trajectories, including associations with cardiovascular and neurological conditions, as well as all-cause mortality. The following sections provide detailed methodological advancements, validation, and clinical implications of this approach.

### Sleep Foundation Model

#### Foundation Model Architecture

**Figure 1.**
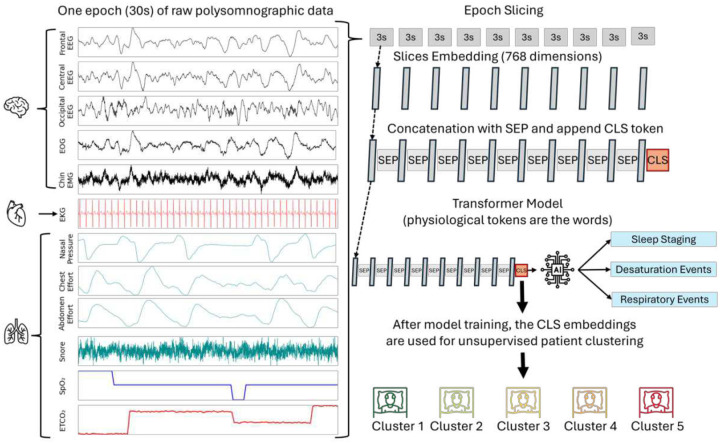
Overall architecture overview. Each 30-second PSG segment is sliced into ten nonoverlapping 3-second segments, which are projected onto a 768-dimensional space using a linear layer. Tokens from all channels are concatenated, split by separator (SEP) tokens, and a classification (CLS) token is appended at the end. The resulting sequence is passed through a Transformer backbone. The CLS embedding output is used to predict technologist-labeled sleep stages, respiratory events, and desaturations, optimized with a cross-entropy loss function. EEG: electroencephalography; EKG: electrocardiography; EMG: electromyography, SpO_2_: oxygen saturation; EtCO_2_: end tidal carbon dioxide.

A transformer-based large language model was adapted for time-series data by replacing its original word embedding layer with a linear projection layer for time-series patches, following a strategy similar to Zhou et al^10^. More specifically, a pre-trained RoBERTa-based model^11^ was fine-tuned on PSG data, employing the architecture illustrated in Figure 1. Unlike the original approach, where some weights remained frozen, all previously trained RoBERTa weights were fine-tuned alongside the newly introduced projection layers and parameters. This adaptation refined the model’s generalized capabilities, tailoring them to the distinctive characteristics of PSG data, including waveforms, time-series structures, and multimodal sensor channels.

To train the model effectively, we used PSG data that captures a wide range of physiological signals during sleep. The dataset included the following channels: C4 (EEG signal from the right central region), F4 (EEG signal from the right frontal region), O2-M2 (EEG signal from the right occipital region), SpO_2_ (oxygen saturation measured via pulse oximetry), EKG (electrocardiogram for electrical heart signal), E1 and E2 (electrooculogram signals from left and right eyes), chin EMG (electromyogram signal from the submental muscle), nasal pressure (airflow measurement via nasal pressure), AIRFLOW (thermal sensor measuring oral airflow), chest and abdomen effort (respiratory effort signals from chest and abdominal movements via inductance plethysmography), snore (vibration sensor measuring snoring activity), and EtCO_2_ (end-tidal carbon dioxide concentration). Each 30-second PSG epoch was divided into ten non-overlapping 3-second segments, which were linearly projected into a 768-dimensional space, resulting in 10 tokens per channel (Figure 1). This time segmentation was selected to mirror the time frame used by clinical technologists. These channels collectively capture neural activity, ocular movements, respiratory patterns, cardiac signals, muscle activity, snoring, oxygen saturation, and carbon dioxide monitoring providing a comprehensive physiological snapshot during sleep.

One advantage of using the CLS token in this architecture (Figure 1) is that it enables the model to condense the complex multi-channel dynamics and relationships of an entire night of sleep into a compact representation while retaining the richness of the physiological data. Specifically, tokens from each channel were concatenated into a single sequence, with a learnable separator token (SEP) and a learnable CLS token appended at the end of the sequence. The CLS token served as a global representation of the input sequence for downstream classification tasks. The sequence was passed through the RoBERTa-based transformer backbone, with the CLS token embedding projected onto separate linear layers for each task: sleep stage classification, respiratory event detection, and desaturation detection.

#### Model Classification Performance

Following training, model performance was evaluated on the testing subset for three classification tasks: 1. sleep stage recognition (non-rapid eye movement sleep stage 1 (N1), N2, N3, and rapid eye movement sleep stage (REM)), 2. respiratory event detection (apnea or hypopnea defined by 3% desaturation or arousal or only 4% desaturation depending on insurance requirements, as this was a clinical sample), and 3. desaturation event detection.

To account for class imbalances, the reported metrics include F1 score and average precision (AP). Both macro- and micro-averaged metrics are provided for the multi-class sleep stage classification. This classification task achieved a macro-averaged F1 of 0.75, a micro-averaged F1 of 0.86, and macro- and micro-averaged AP of 0.62 and 0.76, respectively. These metrics indicate robust performance in the automated, AI-driven labeling of sleep stages and are consistent with previously reported findings from studies that performed sleep stage classification using PSG data, achieving similar levels of performance^12^.

In comparison, respiratory and desaturation classification performance results were slightly lower. Respiratory event classification yielded an F1 score of 0.65 and AP of 0.72, while desaturation event classification exhibited an F1 score of 0.59 and AP of 0.69. Minor differences in F1 and AP scores for these binary tasks likely arise from the labeling approach, wherein each 30-second epoch was labeled as a respiratory or oxygen desaturation event if at least five seconds of the manually labeled annotation overlapped with that epoch. This criterion sometimes caused events to span multiple epochs, diluting per-epoch predictive clarity. Irrespective of this, combined results underscore that our model can robustly and accurately discriminate between sleep stages while also detecting respiratory and desaturation events. Importantly, these classification tasks are intermediate objectives designed to ensure that the model ‘learns’ a physiologically relevant feature space, forming the basis for our main goal of generating embeddings used towards risk group clustering.

### Novel Sleep Profiles

#### Cluster Embeddings into Risk Groups

**Figure 2:**
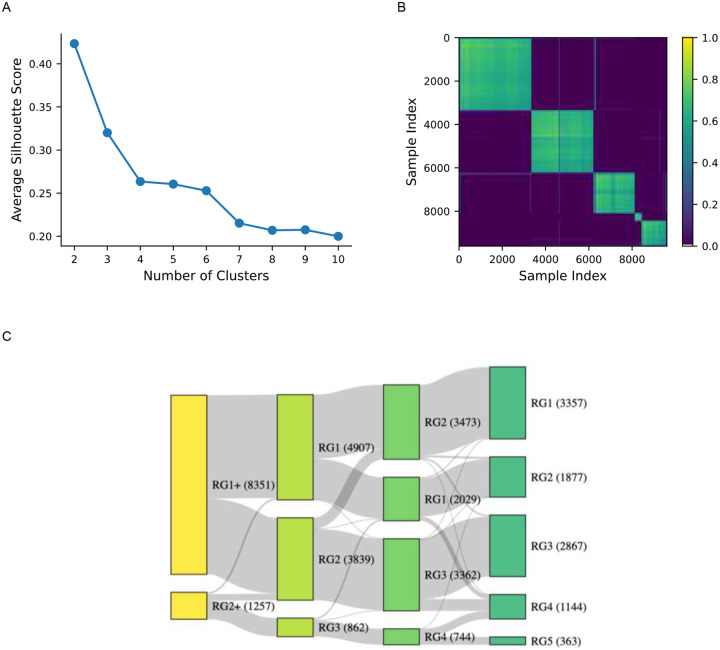
Quantitative assessment of cluster permutations. Cluster definitions shown here use projections on all samples and were calculated using energy distance. The average silhouette score for different numbers of clusters is provided **(A)** with the consensus matrix visualization for five clusters shown in **(B)**. The assignment of sleep study samples to each cluster across the 2-, 3-, 4-, and 5-state solutions is shown in a Sankey flow diagram **(C)** where color indicates the number of clusters per permutation.

After training the foundation model, all PSGs passing quality thresholds were transformed into a high-dimensional embedding space that captured its physiological richness and complexity. Clustering analysis, detailed in the Methods section, was performed on these embeddings using k-means, with several permutations of k-values, distance method, and sampling strategy (Supplementary Figures 1–3). Silhouette score analysis and consensus matrices indicated that five clusters, based on energy distance and utilizing all samples, represented the largest stable solution (Figure 2). The five cluster descriptions were further refined through disease incidence and mortality analyses. Based on clinical outcomes, this process enabled *a posteriori* labeling of risk groups, denoted as RG1 through RG5 (Figure 2). Results from Cox regression and propensity score matching, which will be described in subsequent sections, further supported these assignments.

The five-cluster solution provided the greatest granularity, distinguishing a smaller, high-risk group (RG5) characterized by significant comorbidities and PSG abnormalities consistent with severe sleep disruption. At the other end of the spectrum, RG1 and RG2 represented the lowest-risk groups with minimal abnormalities and comorbidities. Conversely, the more straightforward two-cluster approach isolated a high-risk subgroup, RG2^+^, which largely overlapped with RG5 from the five-cluster solution (Figure 2C). Throughout this paper, we focus primarily on the five-cluster solution for its granularity and the uniqueness of its clusters, while also examining RG2^+^ from the two-cluster solution as it corresponds to RG5 in the five-cluster framework.

#### Associations between Risk Groups and Incidence of Clinical Outcomes

**Figure 3:**
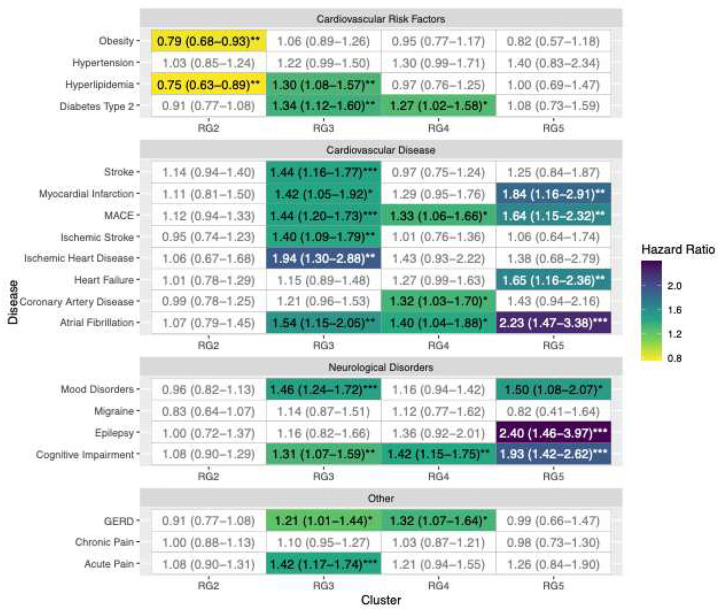
Hazard ratios of disease incidence among risk groups. Hazard ratios (HR) were calculated using all available data for each risk group using RG1 as a reference group due to its ordinal ranking from observed lower incidence of mortality and other adverse health outcomes (hence RG1 is not represented in this figure). Fill color is provided for statistically significant HR values such that darker color indicates higher likelihood of disease and worse outcome. The 95% confidence interval values are included parenthetically, and significance levels are indicated with asterisks: *p<0.05; **p<0.01; and ***p<0.001. Abbreviations: MACE: Major Adverse Cardiovascular Events; GERD: Gastroesophageal Reflux Disease.

Cox proportional hazards models adjusting for demographics and relevant comorbidities revealed a strong monotonic trend for disease incidence when comparing clusters from RG1 to RG5 over 14.5±7.1 year observation period (Figure 3). Across the five-cluster solution, RG5 consistently exhibited the highest hazard ratios (HR) for cardiovascular and neurologic conditions, including myocardial infarction, MACE, heart failure, atrial fibrillation, mood disorders, epilepsy, and cognitive impairment. Notably, RG3 and RG4 also showed elevated risk compared to the reference group (RG1), although to a lesser extent. The associations persisted after propensity-score matching and trimming for outliers (Supplementary Table 1), indicating that the cluster assignments captured meaningful physiological differences not accounted for by simply classifying SDB severity using AHI.

Figure 3 presents HR and 95% confidence intervals (CIs) for incident clinical outcomes, estimated from Cox regression models adjusted for demographic factors and comorbidities, across five risk groups (RG1 to RG5). RG1 represented the lowest risk and lowest mortality, while RG5 represented the highest risk for the development of adverse clinical outcomes. RG1 was used as the reference group due to its high sample size and low-risk profile, making it a stable and representative baseline for comparison (Figure 2 and Supplementary Figure 4). Overall, there was a trend of increasing HRs as risk group number increases, indicating a progressive increase in disease incidence compared to the reference group.

Most associations in RG2 were close to 1 and not statistically significant, suggesting no substantial increase in disease incidence compared to RG1. Notably, hyperlipidemia (HR=0.75; 95% CI 0.63–0.89; p<0.01) and obesity (HR=0.79; 95% CI 0.68–0.93; p<0.01) in RG2 showed significantly lower incidence of these conditions compared to the reference group.

Patients in RG3 exhibited significant increases in association of incidence of several conditions. There was increased association with diabetes type 2 (HR=1.34; 95% CI 1.12–1.60; p<0.01), hyperlipidemia (HR=1.30; 95% CI 1.08–1.57; p<0.01), major adverse cardiovascular events (MACE) (HR=1.44; 95% CI 1.20–1.73; p<0.001), ischemic heart disease (HR=1.94; 95% CI 1.30–2.88; p<0.001), myocardial infarction (HR=1.42; 95% CI 1.05–1.92; p<0.05), stroke (HR=1.44; 95% CI 1.16–1.77; p<0.001), and mood disorders (HR=1.46; 95% CI 1.24–1.72; p<0.001). These findings suggest that RG3 patients have a moderately increased association of both cardiovascular and neurological conditions compared to RG2.

In RG4, the elevated risks persisted for certain diseases. Significant associations were observed for diabetes type 2 (HR=1.27; 95% CI 1.02–1.58; p<0.05), MACE (HR=1.33; 95% CI 1.06–1.66; p<0.05), coronary artery disease (HR=1.32; 95% CI 1.03–1.70; p<0.05), atrial fibrillation (HR 1.40; 95% CI 1.04–1.88; p<0.05), cognitive impairment (HR=1.42; 95% CI 1.15–1.75; p<0.01), and gastroesophageal reflux disease (GERD) (HR=1.32; 95% CI 1.07–1.64; p<0.05). These results indicate that RG4 patients have a continued elevated risk, particularly for cardiovascular diseases and neurologic outcomes.

The highest risk group, RG5, showed the highest magnitude of association with adverse clinical outcomes. Significant associations were noted for MACE (HR=1.64; 95% CI 1.15–2.32; p<0.01), heart failure (HR=1.65; 95% CI 1.16–2.36; p<0.01), myocardial infarction (HR=1.84; 95% CI 1.16–2.91; p<0.01), atrial fibrillation (HR=2.23; 95% CI 1.47–3.38; p<0.001), cognitive impairment (HR=1.93; 95% CI 1.42–2.62; p<0.001), and epilepsy (HR=2.40; 95% CI 1.46–3.97; p<0.001). These findings highlight a substantially increased risk of serious cardiovascular and neurological conditions in RG5 patients.

To assess the robustness of these findings, a sensitivity analysis was conducted using propensity score matching to control for potential confounding variables (described in Methods Section: Statistical Analyses). Supplementary Figure 4 summarizes the 6-year disease-free survival rates among propensity score matched patients across all risk groups. Disease free survival rates decreased progressively from RG1 to RG5 for most conditions, reflecting higher incidence rates in higher risk groups. For instance, the 6-year disease-free survival for MACE was 80.6% in RG1, decreasing to 67.3% in RG5 (p<0.001). Similarly, cognitive impairment survival rates declined from 84.3% in RG1 to 75.2% in RG5 (p<0.01).

Supplementary Table 1 presents HR after propensity score matching. The pattern of increasing association with the clinical outcomes in the higher risk groups remained consistent. Specifically, in RG3, significantly higher HR were observed for hyperlipidemia (HR=1.27; 95% CI 1.05–1.53; p<0.05), diabetes type 2 (HR=1.33; 95% CI 1.11–1.59; p<0.01), MACE (HR=1.35; 95% CI 1.13–1.62; p<0.01), ischemic heart disease (HR=2.01; 95% CI 1.37–2.95; p<0.001), stroke (HR=1.40; 95% CI 1.14–1.72; p<0.01), and mood disorders (HR=1.39; 95% CI 1.18–1.63; p<0.001). In RG4, significant associations were noted for diabetes type 2 (HR=1.38; 95% CI 1.11–1.72; p<0.01), myocardial infarction (HR=1.43; 95% CI 1.03–1.99; p<0.05), atrial fibrillation (HR=1.43; 95% CI 1.04–1.97; p<0.05), and cognitive impairment (HR=1.35; 95% CI 1.08–1.68; p<0.01).

RG5 again demonstrated the highest degree of association with longitudinal clinical outcome development. Significant increased incidence was found for MACE (HR=1.56; 95% CI 1.09–2.24; p<0.05), heart failure (HR=1.53; 95% CI 1.03–2.27; p<0.05), myocardial infarction (HR=1.92; 95% CI 1.19–3.12; p<0.01), atrial fibrillation (HR=1.96; 95% CI 1.24–3.10; p<0.01), cognitive impairment (HR=1.61; 95% CI 1.16–2.24; p<0.01), and epilepsy (HR=1.96; 95% CI 1.16–3.32; p<0.05). These results corroborate the initial findings and confirm that higher risk groups are associated with increased incidence of serious health conditions, even after accounting for confounding factors through propensity score matching. More in depth results for each incident disease are available in Supplementary Table 2 and 3.

#### All-Cause Mortality

**Figure 4:**
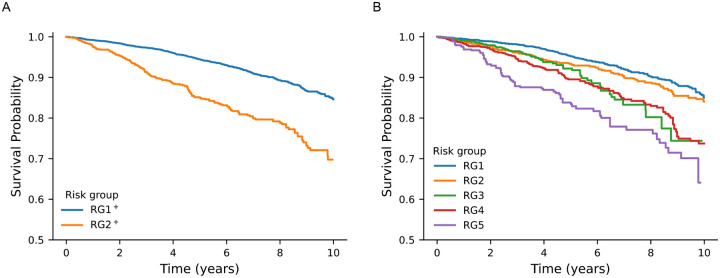
Kaplan-Meier plot for all-cause mortality of propensity matched cases for different risk groups. **(A)** Kaplan-Meier plot for all-cause mortality of propensity-matched cases for the two-cluster solution, RG1^+^ and RG2^+^, with p = 1.2e-42. **(B)** Kaplan-Meier plot for all-cause mortality of propensity-matched cases for the five-cluster solution, where risk groups RG3, RG4, and RG5 are significantly different from RG1 (p=2.1e-6, p=3.9e-24, p=1.7e-31). The risk of all-cause mortality for RG5 is significantly higher than other risk groups. The survival probability observed reflects the ordinal ranking of the risk groups.

The risk groups also showed clear stratification with respect to all-cause mortality. Kaplan-Meier survival curves (Figure 4B) showed that RG5, and to a lesser degree RG4 and RG3, experienced substantially higher mortality rates compared to RG1. This pattern was confirmed with Cox proportional hazards regression (Table 1) that adjusted for age, sex, body mass index (BMI), and comorbidities (hypertension, diabetes type 2, heart failure, atrial fibrillation, coronary artery disease, hyperlipidemia). Additional models that excluded those prescribed continuous positive airway pressure (PAP) therapy or patients under 55 years of age demonstrated consistent findings (Table 1: *Models 5 and 6*, respectively). Notably, these results persisted independent of AHI (Table 1: *Model 4*), illustrating that these novel sleep profiles predicted mortality even after accounting for the influence of traditional classification of mild, moderate, or severe SDB.

Figure 4 presents the survival curves for the risk groups. Panel A shows the Kaplan-Meier curves for the two-cluster solution, highlighting a significant difference in mortality between RG2^+^ and RG1^+^ (p=1.2e-42) where RG2^+^ loosely corresponds to the cohort of RG5 as shown previously (Figure 2C). The survival curves for the five risk groups (RG1 to RG5) after propensity score matching are shown in Figure 4 *Panel B*. RG3, RG4, and RG5 exhibit significant differences compared to RG1 (p=2.1e-6, p=3.9e-24, p=1.7e-31), with increasingly divergent survival patterns and a markedly higher risk of all-cause mortality.

Table 1 presents HR for all-cause mortality across risk groups. Six Cox proportional hazards models were employed, each adjusted for different demographic factors and comorbidities, to conduct sensitivity analyses and evaluate the robustness of the observed associations for all-cause mortality. Multiple model permutations were conducted here for all-cause mortality but were not completed for all other assessed comorbidities due to the expansive computational overhead.

Consistent across all models, there was a progressive increase in mortality from RG2 to RG5. In the fully adjusted Model 4 which accounts for age, sex, BMI, comorbidities, and AHI, HRs for all-cause mortality remained significantly elevated: 1.43 for RG2, 1.54 for RG3, 1.75 for RG4, and 2.38 for RG5 (all p-values < 0.01). The persistence of these findings after controlling for AHI demonstrated that the increased mortality associated with higher-risk groups is independent of traditional SDB severity classification. In fact, a Cox regression analysis performed using the same covariates but replacing RG2 to RG5 with mild, moderate and severe sleep apnea defined by AHI thresholds (mild: ≥5 to <15, moderate: ≥15 to <30, severe: ≥30) showed no increase in disease incidence or all-cause mortality with respect to normal AHI (<5) (Supplementary Table 4).

These results remained robust in models that excluded patients prescribed PAP treatment (Model 5) and those under 55 years of age (Model 6), suggesting that PAP therapy and older age do not substantially alter the observed associations with increased mortality. The consistent pattern of increasing HR point estimates underscores a strong association between higher-risk group classification and increased all-cause mortality, independent of potential confounders, including AHI.

#### Predicting Clusters Using Standard PSG Metrics

We next investigated whether the embedding-based risk groups could be predicted using alternative PSG variables, including commonly used AHI, arousal index, total sleep time, sleep stage percentages, oxygen saturation, and our proposed quantitative definition of “sleep fragmentation.” Herein, we define a metric termed sleep fragmentation calculated as the normalized power in the “fast” frequency range of the hypnogram’s power spectral density (PSD), specifically transitions occurring faster than 10 minutes. For these variables, a gradient boosting classifier (XGBoost^13^) was trained to predict cluster membership for the two-, three-, four-, and five-cluster solutions using combinations of the top 1 and 5 features ranked by importance. Supplementary Table 5 summarizes the classification performance and lists the top 5 features for each solution.

For the two-cluster solution, prediction accuracy exceeded 90% using a single feature related to sleep fragmentation. Accuracy further increased and plateaued at 94% when incorporating the top five PSG-derived features. However, for the more complex three-, four-, and five-cluster solutions, prediction accuracy dropped substantially, even when using multiple features. These findings suggest that while simpler risk group structures can be approximated using standard PSG metrics, the full richness of the foundational embeddings and their ability to capture subtle, multidimensional sleep physiology cannot be fully realized by conventional PSG variables alone.

#### External Validation

Among the clustering solutions tested, the two-risk group approach demonstrated the highest classification accuracy using standard PSG metrics, with sleep fragmentation alone achieving exceptional predictive performance. Given the strong performance of this simplified two-cluster separation, we applied the sleep fragmentation-based classifier to an external population from the geographically diverse, multicenter Sleep Heart Health Study (SHHS) extracted from the National Sleep Research Resource^8^. Patients assigned to the higher-risk cluster demonstrated a significantly greater likelihood of heart failure and mortality, findings observed in both males (p = 0.01 for HF; p<0.001 for mortality) and females (p<0.001 for HF; p<0.001 for mortality) (Supplementary Figure 6).

After adjusting for age and sex using Cox proportional hazards regression, RG2^+^ remained significantly associated with a higher incidence of heart failure (HR=1.52; 95% CI 1.01–2.27; p < 0.05) and mortality (HR=1.72; 95% CI 1.34–2.22; p < 0.001). Notably, consistent with the baseline characteristics of RG5 in our dataset, patients in RG2^+^ had markedly low average total sleep time (182.1 minutes, ~3 hours, with a standard deviation of 37.1 minutes). The external validation supports the robustness of the two-cluster risk separation and highlights the clinical relevance of sleep fragmentation as a simple yet meaningful and expedient measure for identifying high-risk patients.

## DISCUSSION

We developed a foundation model that leverages a large clinical PSG registry of multimodal unstructured sleep signals with structured data from EMR, including details of clinical characteristics. This work introduces key technological innovations by employing time-series modeling and clustering techniques to generate unique “sleep embeddings” that can stratify patients and predict clinical outcomes. By capturing the complex interplay between sleep architecture and respiratory event parameters, our approach identifies distinct phenotypic groups associated with adverse clinical outcomes while accounting for confounding factors. To this end, the model was optimized to simultaneously score sleep stages, respiratory events, and desaturations while also achieving results comparable to previous polysomnographic foundation models^14,15^. Our approach leveraged these techniques to reveal distinct risk groups with clinical interpretability and demonstrate clear incremental risk associations. We address a key priority area identified in the American Thoracic Society Workshop^16^ that promotes using ML and AI to find novel biomarkers to predict cardiovascular and other health outcomes in sleep disorders.

Among the tested clustering solutions, the two-risk-group approach demonstrated the highest classification accuracy when using standard PSG metrics, particularly solely the sleep fragmentation feature defined herein. This makes the two-cluster solution particularly advantageous for generalization to other datasets, as these metrics, including AHI, sleep stages, and respiratory parameters, are routinely calculated during in-laboratory sleep testing, regardless of the data acquisition system. Notably, one of the clusters, labeled RG2^+^, remained relatively consistent across different clustering solutions and corresponds closely to RG5 in the five-cluster approach (Figure 2C). However, for the three-risk group and higher-order solutions, predictive accuracy decreased substantially (Supplementary Table 5). This suggests that more complex risk group solutions based on foundation model embeddings capture nuanced patterns that cannot be easily reproduced using standard PSG metrics alone. Furthermore, when applying the classifier, we developed for the two-cluster solution using standard PSG metrics in the SHHS dataset^17^, we observed similar associations of RG2^+^ from the two-cluster solution with mortality and heart failure, in both sexes. This contrasts with the original SHHS analysis^18^, which identified an association only between severe sleep apnea defined by AHI and heart failure in males alone. This external validation underscored the generalizability of RG2^+^ as a meaningful risk group, suggesting that even basic clustering solutions can be clinically informative while complex solutions provide deeper insights into patient heterogeneity.

This study also highlights the broader limitations of relying solely on AHI as a metric for evaluating SDB presence and severity. While AHI will likely remain an important measure, our findings suggest that additional metrics provide complementary insights into the complexity of SDB. The phenotypic clusters identified in this study align with growing evidence in existing literature that distinct pathophysiological mechanisms underlying SDB extend beyond what AHI captures in different clinical outcomes; these mechanisms led to outcomes including chronic pain^19^, cardiovascular disorders^3^, neurodegenerative pathologies^20^, and all-cause mortality^21^.

A key strength of our study is the use of a large, demographically diverse dataset enriched with minority representation with collection occurring over a decade, containing detailed demographics, and clinical characteristics extracted from the EMR data that was standardized and integrated with PSG information. This comprehensive dataset allowed us to explore the complex relationships between sleep phenotypes and health outcomes with greater precision and granularity. The availability of longitudinal data with mean observation period of 14.5 years further enabled us to investigate long-term associations between the identified risk groups and adverse incident clinical outcomes, such as cardiovascular and neurological diseases. These results have implications for clinical practice and risk stratification and may inform specific treatment approaches. For example, patients in RG5 who demonstrated the highest risk of all-cause mortality may require targeted interventions beyond SDB-targeted therapies. To enhance rigor, we conducted propensity score matching in an attempt to address confounding influences. Furthermore, we conducted external validation of results from this large clinic-based cohort by leveraging a large prospective multicenter population-based cohort, thus supporting reproducibility of the findings.

However, several limitations should be considered. One notable limitation is the lack of objective treatment adherence data which may have influenced clinical outcomes. For example, while PAP prescription information was available, PAP adherence and usage of other therapies for SDB and other sleep disorders were not. Inadequate ascertainment of PAP treatment, however, would be expected to bias findings to the null. Medication usage was not available and may impact the associations discussed. Additionally, while the phenotypic clusters derived from foundation model embeddings demonstrated stronger associations with adverse health outcomes compared to AHI-based classifications, further validation steps are necessary. Further, our findings should be replicated in multiple diverse datasets to ensure their robustness and generalizability. The retrospective nature of the analysis also limits its ability to establish causality, underscoring the need for prospective, case-controlled studies to confirm these results and assess their clinical applicability. Characterizing symptoms and patient reported outcomes with the objective sleep data would be a future area of investigation of clinical relevance and applicability.

In conclusion, our study presents a promising approach to risk stratification in populations undergoing PSG, particularly in the assessment of SDB, leveraging supervised and unsupervised machine learning techniques to extract novel insights from PSG data. The combination of a large dataset and rich patient medical history enabled us to uncover meaningful associations between sleep phenotypes and adverse outcomes. This approach holds promise for improving risk assessment and guiding personalized treatment strategies. Future research should focus on validating these findings prospectively and exploring the integration of this approach into clinical workflows in implementation science paradigms.

## METHODS

### Data Collection

The STARLIT (Sleep Signals, Testing, and Reports Linked to patient Traits)-10K is a retrospective clinical cohort study approved by the Cleveland Clinic Institutional Review Board (IRB#23–409), assembling a comprehensive database of 10,000 in-laboratory PSG studies from the STARLIT Registry^22^, along with patients’ corresponding EMR data. The sleep studies were conducted at the Cleveland Clinic between January 2012 and December 2022. For each visit, Nihon Kohden equipment and Polysmith software were used to collect sleep physiological data, sleep staging information, epoch-by-epoch annotations, and the sleep study report finalized by a board-certified sleep physician. The first 1,000 PSGs were selected randomly, and the remaining 9,000 PSGs were intentionally selected to reflect a broad range of ages, temporal distribution, and enriched minority representation over the entire cohort. Clinical characteristic data were extracted from EMRs using natural language processing from various sources, including hospital admissions, patient encounters, problem lists, referrals, and surgical logs. All-cause mortality was determined by integrating multiple sources, including the EMRs for the most current data, the Ohio Death Index, and the Social Security Death Index. The median duration of medical history available for each patient was 9.4 (IQR: 4.6, 13.6) years before PSG and 4.4 (IQR: 2.0, 5.7) years after testing, for a total of 15.1 (IQR: 9.4, 19.0) years for a mean observation period of 14.5 ± 7.1 years. An overall data description, including the multi-institutional collaboration that uses STARLIT-10K is described in Bilal et al.^23^

### Data Preprocessing

Each PSG contains data from sensors measuring multiple physiological aspects of sleep, including EEG, EOG, EMG, EKG, respiratory airflow, thoracic and abdominal movement, EtCO_2_, snoring, and SpO_2_. The PSG data were preprocessed to remove artifact and ensure consistency across studies. The time-series data were resampled to 128 Hz and preprocessed as described in Brink-Kjaer et al.^24^ Signals were filtered using infinite impulse response (IIR) filters to eliminate artifacts and ensure that signals contained similar spectral content across recordings. The IIR filters were implemented as elliptic filters with an order of 16, a maximum passband ripple of 1 dB, and a minimum stopband attenuation of 40 dB. The cut-off frequencies for the filters were: EEG and EOG: band-pass (0.3–45 Hz); EMG: high-pass (10 Hz); EKG: high-pass (0.3 Hz); nasal pressure: high-pass (0.1 Hz); airflow and plethysmography belts: band-pass (0.1–15 Hz); and blood oxygen saturation: no filtering. All filters were applied forwards and backwards to avoid signal phase distortion. Finally, the signal amplitudes, except blood oxygen saturation, were normalized such that −1 and 1 corresponded to the 5th and 95th percentiles. The blood oxygen saturation was normalized to −1 and 1 corresponding to 60% and 100% saturation.

### Model Training and Embedding Generation

The time-series foundation model was trained to address three primary tasks: sleep stage classification, respiratory event detection which included both apneas and hypopneas, and desaturation event identification. A total of 9,203 PSGs were used for training, with 30 samples forming a validation set and 500 samples set aside as a final test set. The details of PSG inclusion criteria are provided in Supplementary Methods: Data Quality Assessment.

The model was optimized using the Adam optimizer^25^ with a learning rate of 1e-5 and trained for 50 epochs with a batch size of 500 samples. Loss functions were task-specific, using cross-entropy loss for sleep stage classification and binary cross-entropy loss for desaturation and respiratory event detection. The final loss was computed as the average of the task-specific losses to ensure balanced optimization across tasks. The model’s internal representation size was set to 768 which is the default representation dimension from the RoBERTa-base model, with 12 transformer layers contributing to a total of 126 million trainable parameters.

Final sleep embeddings were generated by stacking sequential 30-second epoch representations of the CLS tokens from each sleep study into a final two-dimensional embedding matrix.

### Clustering Method

After training the model, the learned CLS token served as an embedding for each 30-second segment of sleep, creating a two-dimensional representation of the PSG data with 768 rows and columns corresponding to the number of 30-second epochs in each recording. Consequently, the embedding dimensions varied between samples due to differences in PSG duration. These differences precluded the direct application of k-means clustering. To address this and compress the feature space, each sample was projected onto all other samples by calculating pairwise distances between its embedding and the embeddings of all other samples. Distances were computed for corresponding rows of the embeddings (representing the time dimension) and averaged across the embedding dimension to produce a single distance value for each pair. This process generated a feature vector for each sample, where each element represented its distance to another sample. These feature vectors were then used as input for k-means clustering.

Given the variability in PSG duration, distance metrics capable of comparing distributions with differing numbers of samples were required. Metrics such as energy distance^26^ and Earth mover’s distance,^27^ both of which are well-suited for comparing distributions of varying sizes, were evaluated. Additionally, the analysis included projections restricted to just the test samples to assess the effect of limiting the sample space.

Clustering performance was assessed using silhouette scores and consensus matrices. The silhouette score quantified the cohesion and separation of clusters, with higher scores indicating better-defined groups. The consensus matrix was generated through subsampling, where 80% of samples and features were randomly selected in 100 iterations. For each pair of samples, the matrix recorded the proportion of iterations in which the samples were assigned to the same cluster. To facilitate interpretation, the consensus matrix was ordered using hierarchical clustering with the Ward criterion,^28^ grouping samples based on their co-clustering frequency to visually highlight stable cluster structures.

Supplementary Figure 1 presents the silhouette scores and consensus heatmaps for clustering based on energy distance applied to all samples, the version selected for subsequent analyses in the study. The heatmaps reveal a clear block-diagonal structure up to five clusters, indicating stable and well-defined groupings. Beyond five clusters, this structure deteriorates, suggesting reduced clustering quality.

A sensitivity analysis was conducted by varying the distance metric and projection size. Supplementary Figure 2 presents the analysis using energy distance with projections limited to test samples, while Supplementary Figure 3 shows the results for Earth mover’s distance applied to test sample projections.

Supplementary Figure 5 illustrates the sample assignment across the three 5-cluster solutions, highlighting the robustness of the five-cluster solution to variations in distance metric and projection size.

These results demonstrate that using energy distance across all samples provides robust clustering, as evidenced by the stable block-diagonal consensus structure for up to five clusters and consistently high silhouette scores. This clustering configuration was subsequently used for further analyses throughout the study.

### Statistical Analyses

Baseline characteristics were compared between risk groups using chi-square tests for categorical variables, with Bonferroni corrections for pairwise comparisons on significant results (p < 0.05). Continuous characteristics were analyzed using Welch’s ANOVA, with significant differences further examined via pairwise *t*-tests with Bonferroni correction. Logistic regression was used to assess associations between total sleep time on the PSG and baseline medical history, adjusting for age, BMI, sex, and relevant comorbidities (Supplementary Table 6).

Survival time was defined as the duration from the date of baseline PSG until the date of death or, for censored cases, the date of last follow-up as recorded in the EMR. Cox proportional hazards regression was used to examine the association between demographic variables (age, sex, BMI), comorbidities, AHI, and clustered risk groups with time-to-event outcomes for various diseases. Relevant comorbidities (Supplementary Table 6) were included for each disease, and only patients with at least 5 years of prior medical history were considered, excluding those with prior occurrences of the disease. The proportional odds assumption was met for all models.

A sensitivity analysis was conducted by performing a second Cox regression on weighted propensity score-matched data to evaluate whether the results of the initial analysis were influenced by residual confounding or imbalances in baseline characteristics. Propensity scores were estimated using multinomial logistic regression to control for confounding across risk groups using age, BMI, sex, relevant comorbidities (Supplementary Table 6), and number of years of medical history available before sleep testing. Propensity weights were calculated as stabilized inverse propensity scores^29^ and were trimmed at the 5th and 95th percentiles to minimize bias introduced by extreme values. Kaplan-Meier analysis estimated disease-free survival probabilities at 2, 4, and 6 years, with log-rank tests comparing survival rates between the reference and other risk groups.

## Supplementary Material

1

## Figures and Tables

**Table 1:** Hazard ratios of all-cause mortality among patients from different risk groups

Model	No samples	No events	RG2	RG3	RG4	RG5
**1**	7043	544	0.92 (0.72–1.17)	1.69 (1.29–2.23)***	3.11 (2.46–3.92)***	4.83 (3.60–6.50)***
**2**	7043	544	1.54 (1.20–1.98)***	1.65 (1.25–2.16)***	1.84 (1.45–2.33)***	2.73 (2.02–3.69)***
**3**	7043	544	1.47 (1.14–1.88)**	1.52 (1.16–2.00)**	1.67 (1.31–2.11)***	2.16 (1.59–2.95)***
**4**	7043	544	1.43 (1.11–1.84)**	1.54 (1.17–2.03)**	1.75 (1.37–2.23)***	2.38 (1.73–3.28)***
**5**	3700	299	1.32 (0.95–1.82)	1.66 (1.14–2.40)**	1.78 (1.28–2.47)***	1.76 (1.12–2.78)*
**6**	1569	523	1.53 (1.15–2.04)**	1.47 (1.08–2.01)*	1.67 (1.30–2.15)***	1.75 (1.24–2.49)**

Model 1: without adjustment; 2: adjusted for age, sex and body mass index (BMI); 3: adjusted for age, sex, BMI and comorbidities (hypertension, diabetes type 2, heart failure, atrial fibrillation, coronary artery disease, hyperlipidemia); 4: adjusted for age, sex, BMI, comorbidities and apnea hypopnea index (AHI); 5: adjusted for age, sex, BMI, comorbidities and excluding patients for which PAP therapy was prescribed; 6: adjusted for age, sex, BMI and comorbidities excluding patients less than 55 years of age.

## Data Availability

Data are available upon request and can be shared in accordance with Institutional Review Board and Data User Agreement limitations.
