# Supplementary material for: A Foundation Model for Sleep-Based Risk Stratification and Clinical Outcomes
Source: Res Sq. 2025 Apr 10:rs.3.rs-6307069. Preprint. [Version 1] doi: 10.21203/rs.3.rs-6307069/v1 (PMC12036469; doi:10.21203/rs.3.rs-6307069/v1)
Supplement: 1 [file NIHPPRS6307069V1-supplement-1.pdf]

SUPPLEMENTARY RESULTS

Baseline analysis of novel risk groups

Supplementary Table 7 presents the demographic and clinical characteristics, along with the sleep parameters defined in *Supplementary Table 8*, for patients across five risk groups, RG1 to RG5. The data are organized under several subheadings: Sociodemographics, Cardiovascular Risk Factors, Cardiovascular Disease, Neurological Disorders, Other Medical History, PSG results, Alternative Metrics, and Other measures.

Risk Group 1 (RG1) had the largest sample size (n=3,357) with a mean age of 50.8 years. This group had a relatively balanced sex distribution, with 52.6% males. The prevalence of cardiovascular risk factors such as hypertension (59.8%), hyperlipidemia (53.5%), and type 2 diabetes (32.7%) was moderate compared to other groups. PSG results indicated mild SDB, with an average AHI of 12.4 events/hour and an arousal index of 23.8 events/hour. This group also showed moderate levels relative to other RGs of sleep fragmentation and hypoxic burden.

Risk Group 2 (RG2) consisted of younger patients with a mean age of 44.0 years and the lowest percentage of males (37.6%). This group exhibited the lowest prevalence of cardiovascular risk factors: hypertension (47.5%), hyperlipidemia (37.8%), and type 2 diabetes (24.7%). PSG findings revealed the mildest SDB of all groups, with AHI of 5.4 events/hour, and the lowest arousal index

(19.3 events/hour). Neurological disorders such as migraine (19.7%) and mood disorders (50.9%) were relatively more prevalent in this group.

Risk Group 3 (RG3) had patients with a mean age of 49.7 years and an intermediate sex distribution (48.7% males). This group showed higher prevalence of mood disorders (56.6%) and chronic pain (44.8%). Cardiovascular risk factors were similar to RG1, with hypertension present in 59.7% of patients. PSG results indicated mild SDB with an AHI of 11.2 events/hour and an arousal index of 22.7 events/hour.

Risk Group 4 (RG4) included older patients with a mean age of 58.6 years and the highest percentage of males among larger groups (60.8%). This group exhibited the highest prevalence of cardiovascular risk factors: hypertension (75.6%), hyperlipidemia (65.2%), and type 2 diabetes (41.8%). Cardiovascular diseases were more common, with heart failure present in 18.4% and atrial fibrillation in 15.3% of patients. PSG results showed increased SDB severity relative to lower risk groups, with an AHI of 22.7 events/hour and a significantly higher arousal index of 41.5 events/hour, suggesting more fragmented sleep.

Similar to RG4, Risk Group 5 (RG5), the smallest group (n=363), had patients with high mean age (58.6 years) and the highest proportion of males (66.4%). This group demonstrated the most severe PSG abnormalities, with the highest AHI (37.3 events/hour) and arousal index (60.8 events/hour), suggesting severe SDB and sleep fragmentation. Cardiovascular diseases were most prevalent in this group, with heart failure in 21.8% and atrial fibrillation in 17.9% of patients. Despite severe PSG findings, the prevalence of some neurological disorders, such as mood disorders (51.0%), was similar to other groups.

To further evaluate the associations between risk groups and various diseases, *Supplementary Tables 9* and *10* provide the odds ratios of comorbidities for patients in risk groups RG2 to RG5 relative to RG1, derived from logistic regression analyses adjusted for age, BMI, and relevant comorbidities (as outlined in *Supplementary Table 6*). These findings largely corroborate the observations from the baseline table, with the exception of RG3, which was significantly associated with increased odds for all the diseases examined. This may be attributed to a strong age effect, given the relatively younger age of individuals in this group.

In summary, the risk groups exhibited a gradient of increasing age, male predominance, cardiovascular risk factors, and severity of SDB from RG2 to RG5. RG2 was characterized by younger age, a lower prevalence of cardiovascular risks, and milder PSG findings. RG4 and RG5 included older patients with higher cardiovascular morbidity and more severe PSG abnormalities, reflecting a higher risk profile. RG1 and RG3 shared similar PSG characteristics; however, RG3 distinguished itself by being associated with a significantly higher number of comorbidities after adjusting for demographic factors.

SUPPLEMENTARY METHODS

Data Quality Assessment

Sample records involving study type that was not manually confirmed as PSG were excluded. This exclusion encompassed split studies, home sleep apnea tests, multiple sleep latency tests, maintenance of wakefulness tests, and other non-PSG studies in which sleep was intentionally or artificially altered.

Data quality assessment for the 10,000 files was performed using data from six referenced channels. In an ideal setup, odd-numbered channels, positioned on the left side of the scalp, were referenced by 'M1', while even-numbered channels, positioned on the right side, were referenced by 'M2'. In rare cases of reference issues, the reference from the opposite side was employed. The electroencephalography (EEG) channels utilized were 'F3', 'F4', 'C3', 'C4', 'O1', and 'O2'[30].

A high-pass filter with a passband frequency of 1 Hz and a notch filter with a stop-band of 57.5–62.5 Hz was applied to all recordings using a zero-phase forward and reverse digital filter. Waveforms in each channel were divided into 1-second epochs, and each epoch was checked for artifacts using a previously validated machine learning method by Levitt et al. [31]. Three separate artifact detection scans were applied to each file, corresponding to the three non-rapid eye movement sleep stages: N1, N2, and N3. Rapid eye movement (REM) sleep was excluded due to the presence of ocular artifacts. Each scan started at the first occurrence of the respective sleep stage in the file and continued for a duration of two minutes.

In each sleep stage, the percentage of clean files was determined based on the artifact detection algorithm's results. A file was labeled as clean if it had at least four clean channels out of the six EEG channels, with each channel considered clean if it had at least 60% artifact-free data. The percentages of clean files for N1, N2, and N3 sleep stages were 90%, 88%, and 91%, respectively. A total of 266 files that were not artifact-free in all three sleep stages were excluded.

Polysomnogram Features

The aggregated PSG metrics were extracted from the structured HTML format of the final sleep study report submitted by the provider to the EMR. The metrics parsed from the original file include total and REM AHI, arousal index, obstructive apneas, central apneas, hypopneas, mean oxygen saturation, minimum and maximum SpO2, sleep time with oxygen saturation less than 90% SpO_2, maximum $EtCO_2$, percentage of sleep time in each sleep stage, total sleep time, total REM time, and snoring. All conventional PSG variables were computed after scoring by Nihon Kohden's Polysmith 12 software. To enrich the analyses, the following alternative metrics were computed: sleep apnea-specific hypoxic burden,[32] sleep apnea-specific pulse-rate response (Delta HR),[33] lung to finger circulation time,[34] and sleep fragmentation, which is defined herein and calculated as the normalized

power in the "fast" frequency range of the hypnogram's power spectral density (PSD), specifically transitions occurring faster than 10 minutes.

Providers are instructed to remove text variables reporting zero values in the final PSG report if the value was not measurable. This may result in missing values that should be zero. With regard to data cleanliness and pre-processing, missing values were imputed where appropriate to address clinical data incompleteness. The following corrections were applied: sleep time with oxygen saturation $<O_2$ under 90% was set to 0 if the minimum oxygen saturation was above 90%. If the central apnea index (CAI) was zero, the number of central apneas was set to zero. To ensure accuracy, the reported AHI total was validated by recomputing the sum of individual counts of respiratory events (obstructive apneas, central apneas, mixed apneas, and hypopneas) based on total sleep time. In case of missing respiratory events and AHI total was zero, individual respiratory events counts were set to zero. When total sleep time was zero, the AHI total was set to N/A, as no sleep was observed.

Disease Vocabulary and Comorbidities

To define the disease vocabulary used in our analyses, we curated a comprehensive list of clinically significant conditions with their respective diagnosis code (ICD-10) that are relevant to SDB, sleep architecture, and associated health risks (*Supplementary Table 11)*. This vocabulary included disease outcomes: type 2 diabetes, hypertension, hyperlipidemia, heart failure (HF), atrial fibrillation (AF), migraine, mood disorders, cognitive impairment, chronic obstructive pulmonary disease (COPD), chronic pain, gastroesophageal reflux disease (GERD), chronic insomnia, and major adverse cardiovascular events (MACE) which included HF, myocardial infarction, coronary artery disease (CAD), coronary artery bypass grafting, and stroke. Mood disorders included depression, anxiety, bipolar disorder, post-traumatic stress disorder, and related conditions. Cognitive impairment included cognitive deficits, amnesia, and dementia-related conditions such as Alzheimer's, Parkinson's, Lewy body disease, and vascular dementia. Chronic pain included chronic pain syndrome and fibromyalgia. These conditions were identified from diagnoses recorded in the Electronic Health Record (Epic®) that appeared at least once in our cohort. They were selected based on both established and hypothesized relationships with sleep physiology and their relevance to long-term health outcomes, with the goal of providing a broad view of how sleep-related risk factors may influence diverse health domains.

For each disease outcome, we individually selected covariates to adjust for relevant confounders that could potentially affect the association between the risk groups and outcomes (Supplementary Table 12). All models included obesity as a covariate, highlighting the importance this condition has in cardiovascular and neurologic diseases[35]. Covariate selection was based upon biological plausibility of factors considered to be confounding influences in the respective models. **Type 2 diabetes** was modeled with hypertension as a covariate, reflecting the well-known role this factor plays in the development and progression of diabetes[36]. **Hypertension** was modeled with diabetes, AF,[37] HF ,

CAD,[38] and hyperlipidemia, [39] acknowledging the multifactorial nature of hypertension and its associations with both metabolic and cardiovascular conditions. **Hyperlipidemia** was modeled with hypertension,[39] diabetes,[40] AF,[41] HF, [42] and CAD,[43] recognizing the intertwined relationships between lipid metabolism and these cardiovascular risk factors. **HF** was modeled with hypertension,[44] diabetes,[45] AF,[46] CAD,[47] and hyperlipidemia,[42] as these factors collectively contribute to the development and worsening of HF. **AF** was modeled with hypertension,[37] diabetes,[48] HF,[46] CAD,[49] and hyperlipidemia,[41] given the strong associations between these conditions and the risk of arrhythmias. **Migraine** was modeled with mood disorders, reflecting the interplay between these factors in the pathophysiology of migraine and sleep disorders.[50] **Mood disorders** were modeled with chronic pain, recognizing how these conditions are often co-occurring and can exacerbate each other's impact on mental and physical health.[51] **Cognitive impairment** was modeled with mood disorders, as this factor may be implicated in the progression of cognitive decline.[52,53] **COPD** was modeled with HF, as obesity and HF can be major contributors to the development and severity of COPD.[54] **Chronic pain** was modeled with mood disorders, emphasizing how chronic pain often coexists with these conditions, influencing the overall health burden.[51] **GERD** was modeled with hypertension and diabetes, reflecting the well-documented associations between these conditions and the development of reflux. **Chronic insomnia** was modeled with chronic pain[55] and mood disorders,[56] highlighting complex interactions that contribute to the persistence of insomnia.[55,56] **MACE** was modeled without obesity and with hypertension, diabetes, AF, and hyperlipidemia, to best capture the risk factors pertinent to these serious outcomes. This tailored approach ensured that each model accounts for disease-specific confounders, providing a robust and nuanced framework for assessing the relationship between the identified risk groups and a wide range of clinically meaningful health outcomes.

Supplementary Figures

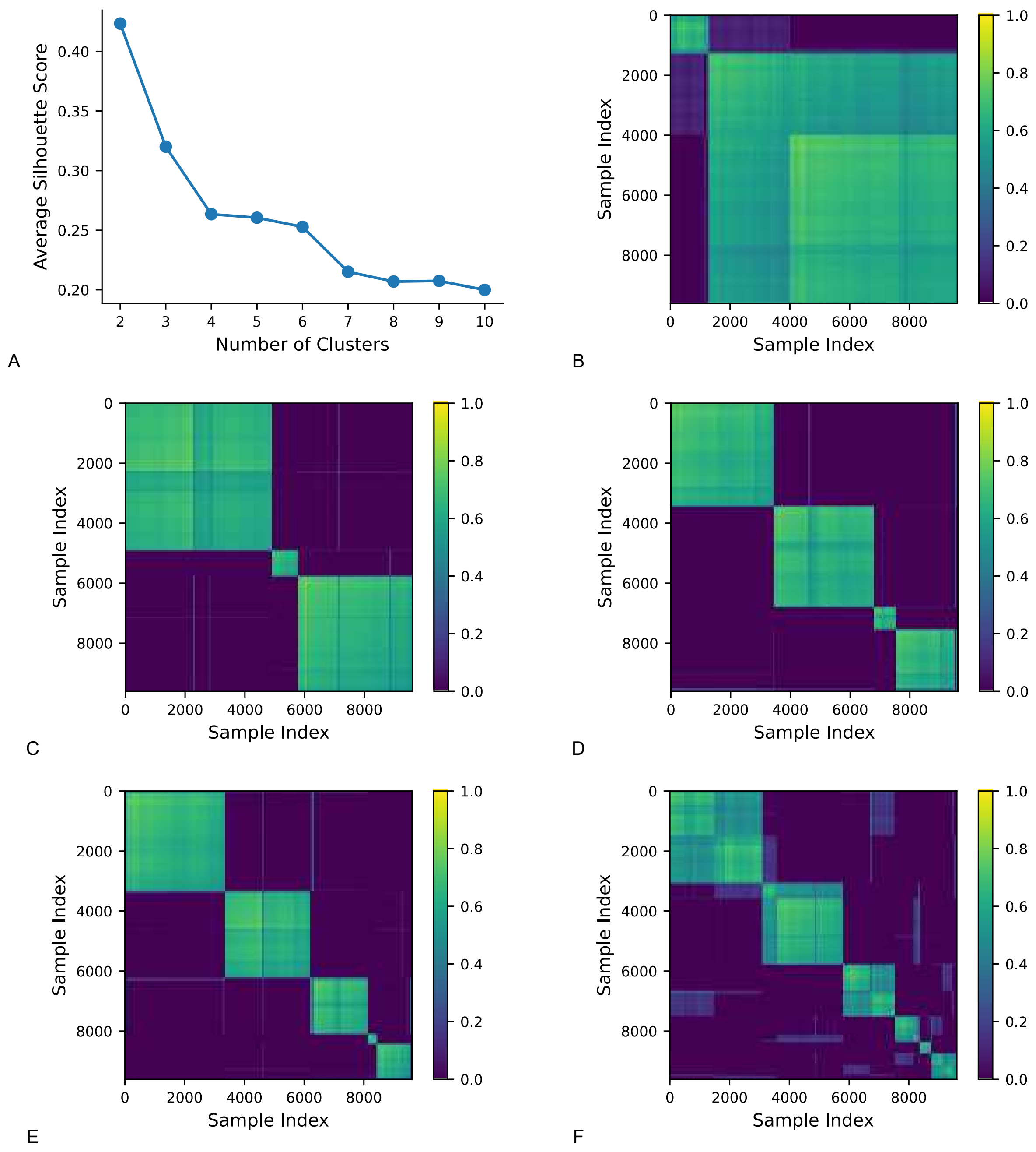

Supplementary Figure 1: Sensitivity analyses of different numbers of clusters derived using energy distance and all samples projection. Clusters use projections on all samples using the energy distance. (A) Silhouette score for different number of clusters, consensus matrices visualization for 2 (B), 3 (C), 4 (D), 5 (E) and 6 (F) clusters. Note panel A and E are reproduced here from Figure 2 to show all k-values and enable a side-by-side comparison.

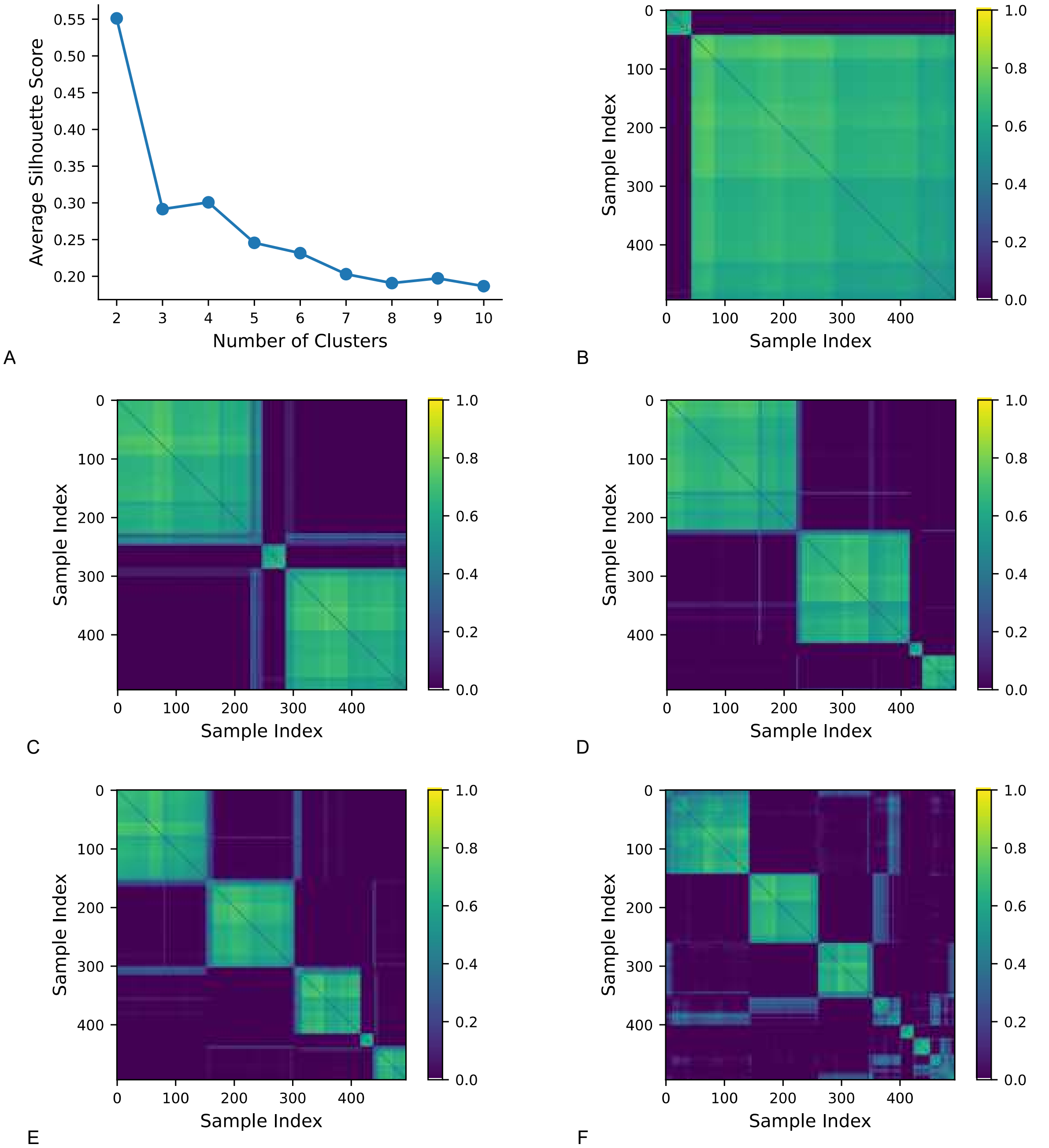

Supplementary Figure 2: Sensitivity analyses of different numbers of clusters derived using energy distance and test samples projection. Clusters use projections on test samples using the energy distance. (A) Silhouette score for different number of clusters, consensus matrices visualization for 2 (B), 3 (C), 4 (D), 5 (E) and 6 (F) clusters

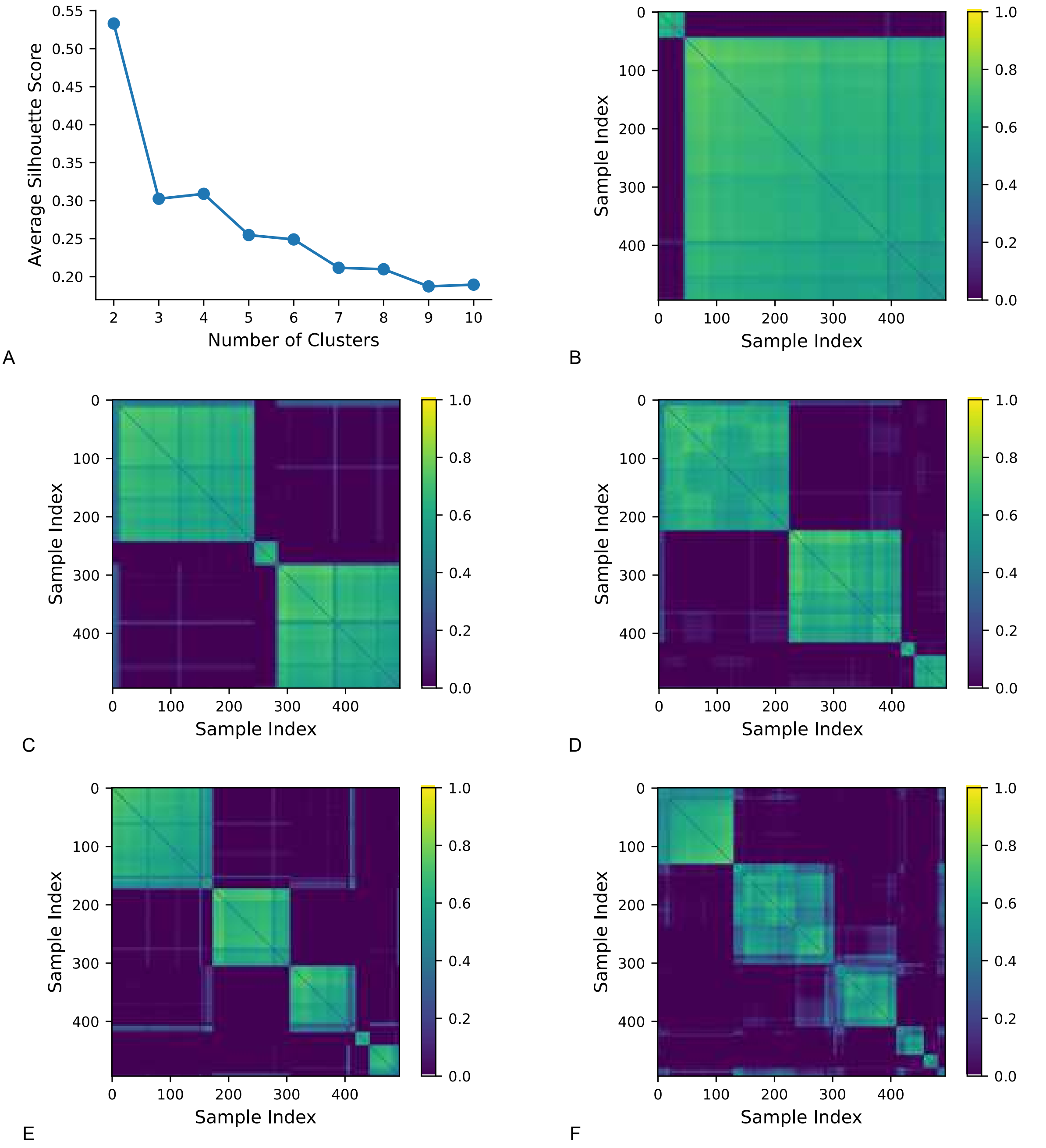

Supplementary Figure 3: Sensitivity analyses of different numbers of clusters derived using Earth mover's distance and test samples projection. Clusters use projections on test samples using the Earth mover's distance. (A) Silhouette score for different numbers of clusters, consensus matrices visualization for (B) two, (C) three, (D) four, (E) five, and (F) six clusters.

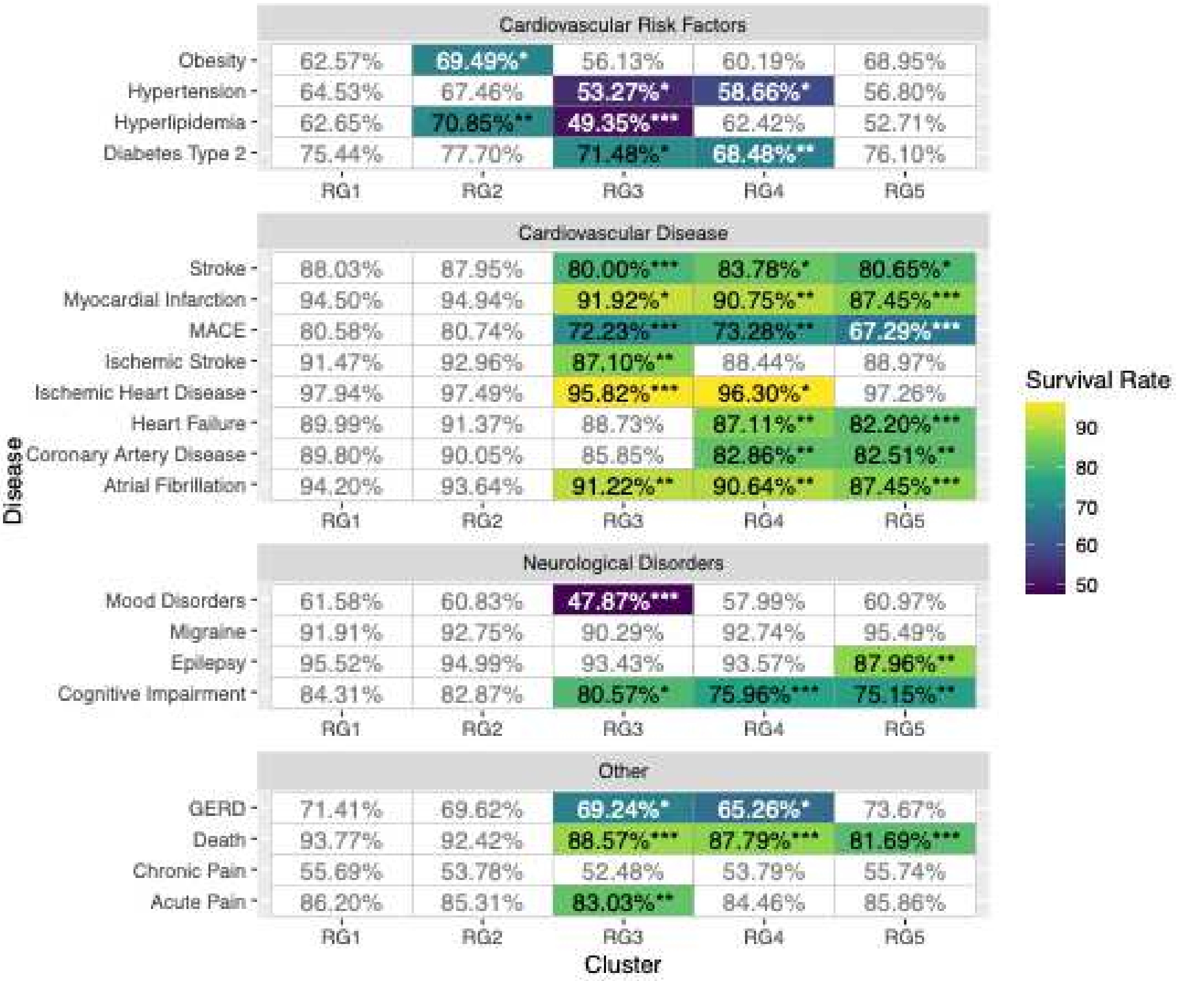

Supplementary Figure 4: Summary of 6-year disease free survival among propensity score matched patients from different risk groups. Survival rate was calculated after six years from the date of baseline PSG. Fill color is provided for statistically significant survival rates with weighted propensity score matching such that darker color indicates worse outcome (lower survival due to disease indicated on y-axis). Significance levels are indicated with asterisks: *p<0.05; **p<0.01; and ***p<0.001. Abbreviations: MACE: Major Adverse Cardiovascular Events; GERD: Gastroesophageal Reflux Disease.

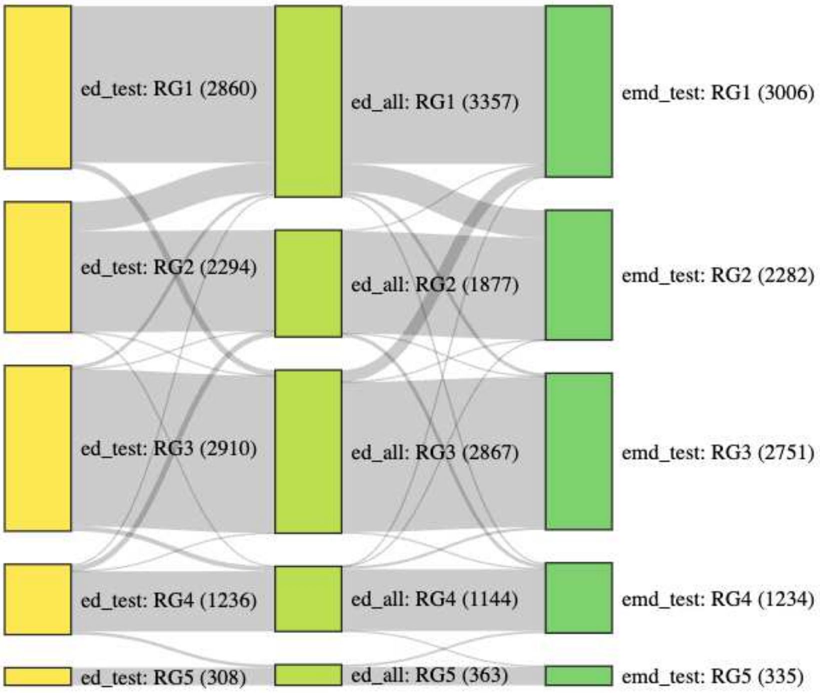

Supplementary Figure 5: Sankey diagram for the sample assignment between different five cluster solutions. The three columns represent the different five-cluster solutions from *Supplementary figures 1 – 3*

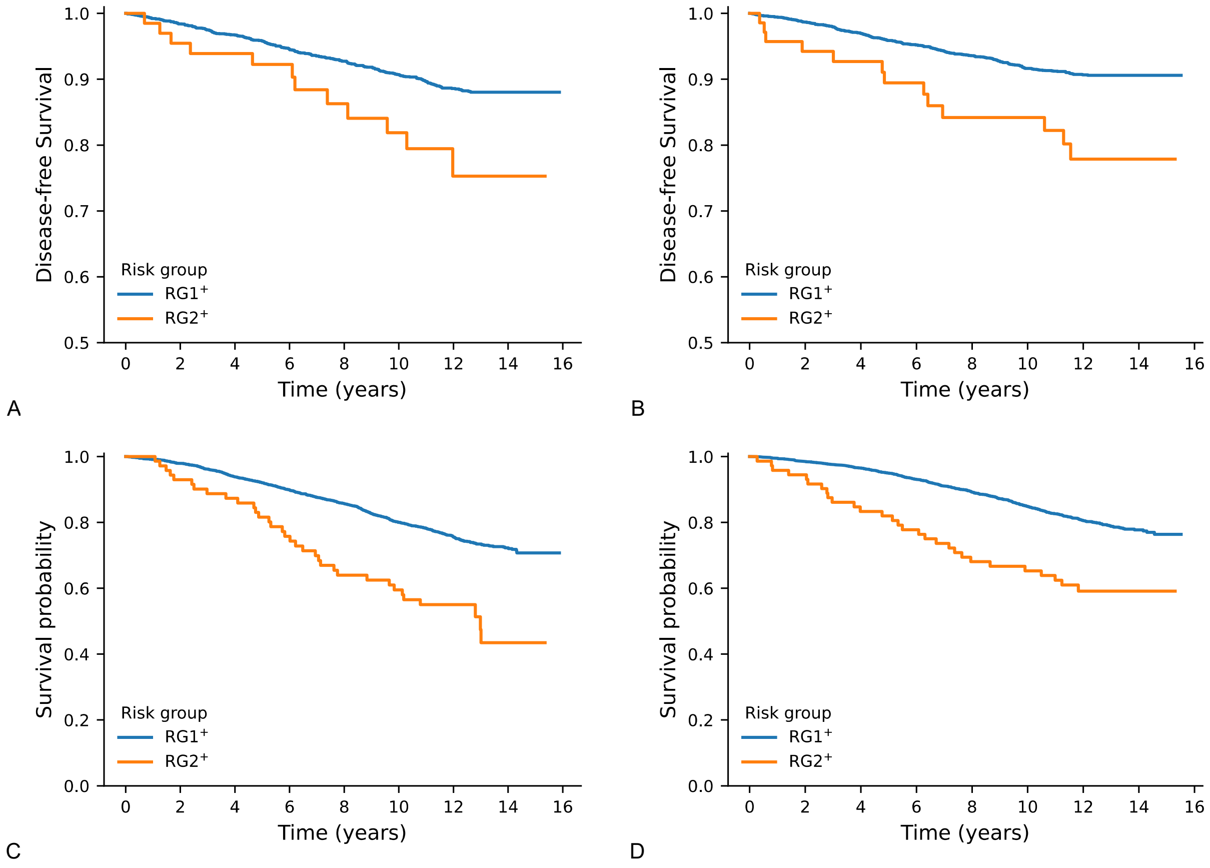

Supplementary Figure 6: Kaplan-Meier plots of risk groups RG1⁺ and RG2⁺ from the two-cluster solution applied to the Sleep Heart Health Study data. Congestive heart failure-free survival plots are shown in panel A for males (p = 0.0103) and panel B for females (p = 0.0008) for risk groups $RG1^+$ and $RG2^+$ of the two-cluster solution applied to the data from the Sleep Heart Health Study. Survival plots for all-cause mortality are shown in panels C for males (p = 3.8e-07) and panel D for females (p = 1.7e-06).

Supplementary Tables

Supplementary Table 1: Hazard ratios of incident diseases and all-cause mortality among patients from different risk groups after propensity score matching

Disease	RG2	RG3	RG4	RG5
**Cardiovascular Risk Factors**				
**Hypertension**	1.08 (0.90-1.30)	1.16 (0.94-1.43)	1.26 (0.94-1.69)	1.31 (0.79-2.16)
**Hyperlipidemia**	0.74 (0.63-0.88)***	1.27 (1.05-1.53)*	1.02 (0.79-1.32)	1.12 (0.77-1.63)
**Diabetes Type 2**	0.93 (0.79-1.10)	1.33 (1.11-1.59)**	1.38 (1.11-1.72)**	1.05 (0.70-1.57)
**Obesity**	0.79 (0.67-0.92)**	1.10 (0.93-1.30)	0.82 (0.66-1.02)	0.90 (0.61-1.33)
**Cardiovascular Disease**				
**MACE**	1.12 (0.95-1.33)	1.35 (1.13-1.62)***	1.23 (0.96-1.57)	1.56 (1.09-2.24)*
**Heart Failure**	1.03 (0.82-1.29)	1.07 (0.83-1.36)	1.23 (0.93-1.62)	1.53 (1.03-2.27)*
**Myocardial Infarction**	1.17 (0.88-1.55)	1.42 (1.06-1.90)*	1.43 (1.03-1.99)*	1.92 (1.19-3.12)**
**Ischemic Heart Disease**	1.05 (0.68-1.61)	2.01 (1.37-2.95)***	1.40 (0.86-2.30)	1.28 (0.56-2.97)
**Coronary Artery Disease**	1.03 (0.83-1.28)	1.15 (0.91-1.44)	1.29 (0.98-1.70)	1.44 (0.93-2.22)
**Atrial Fibrillation**	1.12 (0.85-1.47)	1.35 (1.02-1.80)*	1.43 (1.04-1.97)*	1.96 (1.24-3.10)**
**Stroke**	1.12 (0.92-1.35)	1.40 (1.14-1.72)**	0.97 (0.75-1.27)	1.31 (0.87-1.95)
**Ischemic Stroke**	0.95 (0.75-1.21)	1.32 (1.03-1.68)*	1.01 (0.74-1.38)	1.02 (0.61-1.70)
**Neurological Disorders**				
**Mood Disorders**	1.03 (0.88-1.21)	1.39 (1.18-1.63)***	1.16 (0.95-1.43)	1.24 (0.89-1.73)
**Migraine**	0.81 (0.62-1.06)	1.16 (0.88-1.53)	1.06 (0.75-1.51)	0.75 (0.40-1.40)
**Cognitive Impairment**	1.13 (0.95-1.35)	1.17 (0.96-1.42)	1.35 (1.08-1.68)**	1.61 (1.16-2.24)**
**Epilepsy**	1.07 (0.78-1.47)	1.09 (0.77-1.56)	1.28 (0.86-1.92)	1.96 (1.16-3.32)*
**Other**				
**GERD**	1.05 (0.89-1.25)	1.20 (1.00-1.43)*	1.36 (1.10-1.69)**	0.92 (0.62-1.37)
**Chronic Pain**	1.03 (0.91-1.16)	1.06 (0.92-1.22)	0.99 (0.84-1.18)	0.98 (0.74-1.29)

**Acute Pain**	1.06 (0.88-1.27)	1.36 (1.12-1.66)**	1.14 (0.89-1.46)	1.08 (0.71-1.62)
**Death**	1.49 (1.19-1.87)***	1.54 (1.19-2.00)**	1.67 (1.28-2.19)***	2.49 (1.77-3.50)***

Significance levels: *p<0.05; **p<0.01; ***p<0.001

Supplementary Table 2: Cox regression analysis results for each disease

See attached file: cox_summary_results.xlsx

Supplementary Table 3: Propensity score matching analysis for each disease

See attached file: propensity_analysis_results.xlsx

Supplementary Table 4: Hazard ratios of incident diseases and all-cause mortality among patients from different AHI categories

Disease	AHI Mild	AHI Moderate	AHI Severe
**Cardiovascular Risk Factors**			
**Hypertension**	0.89 (0.75-1.06)	0.91 (0.72-1.14)	1.00 (0.75-1.32)
**Diabetes Type 2**	0.97 (0.83-1.13)	0.90 (0.74-1.09)	1.19 (0.96-1.49)
**Hyperlipidemia**	1.26 (1.07-1.48)**	1.32 (1.07-1.62)**	1.29 (1.01-1.66)*
**Obesity**	1.21 (1.04-1.41)*	0.94 (0.77-1.14)	1.31 (1.06-1.63)*
**Cardiovascular Disease**			
**MACE**	0.84 (0.72-0.99)*	0.74 (0.61-0.90)**	0.76 (0.61-0.97)*
**Heart Failure**	0.83 (0.67-1.02)	0.65 (0.50-0.84)**	0.80 (0.60-1.07)
**Myocardial Infarction**	0.96 (0.74-1.25)	0.79 (0.58-1.09)	1.20 (0.85-1.69)
**Ischemic Heart Disease**	1.31 (0.90-1.91)	0.91 (0.58-1.45)	0.98 (0.58-1.66)
**Coronary Artery Disease**	1.02 (0.83-1.26)	0.91 (0.71-1.16)	1.00 (0.75-1.33)
**Atrial Fibrillation**	0.99 (0.77-1.27)	0.75 (0.55-1.02)	0.76 (0.53-1.10)
**Stroke**	0.78 (0.65-0.93)**	0.79 (0.63-0.97)*	0.75 (0.57-0.97)*
**Ischemic Stroke**	0.87 (0.70-1.08)	0.78 (0.60-1.02)	0.68 (0.49-0.96)*
**Neurological Disorders**			
**Mood Disorders**	0.95 (0.82-1.10)	0.83 (0.70-0.99)*	0.79 (0.64-0.98)*
**Migraine**	1.18 (0.93-1.49)	1.07 (0.78-1.46)	0.92 (0.61-1.41)
**Cognitive Impairment**	0.87 (0.74-1.02)	0.71 (0.58-0.87)***	0.70 (0.54-0.89)**
**Epilepsy**	0.89 (0.67-1.17)	0.65 (0.45-0.95)*	0.59 (0.36-0.95)*
**Other**			
**GERD**	0.95 (0.81-1.10)	0.81 (0.67-0.99)*	0.73 (0.58-0.93)*
**Chronic Pain**	0.93 (0.83-1.04)	0.86 (0.74-0.99)*	0.79 (0.66-0.95)*
**Acute Pain**	0.92 (0.78-1.09)	0.91 (0.74-1.13)	0.73 (0.55-0.97)*
**Death**	0.71 (0.58-0.87)**	0.52 (0.40-0.68)***	0.77 (0.58-1.02)

Disease abbreviations: AHI: apnea hypopnea index, MACE: Major Adverse Cardiovascular Events; GERD: Gastroesophageal Reflux Disease

Significance levels: *p<0.05; **p<0.01; ***p<0.001

Values are Hazard Ratio (95% Confidence Interval)

Supplementary Table 5: Classification of risk groups using standard sleep measures

Model accuracy	2-cluster solution	3-cluster solution	4-cluster solution	5-cluster solution
XGB w/ top-1 features	93.0%	54.6%	41.6%	41.7%
XGB w/ top-5 features	94.1%	67.8%	56.3%	56.2
Top-5 features	Sleep fragmentation<br>Total sleep time<br>AHI total<br>Total REM time<br>Total NREM time	Sleep fragmentation<br>Total sleep time<br>Hypoxic burden<br>Total N3 time<br>AHI total	Sleep fragmentation<br>Total sleep time<br>AHI total<br>Hypoxic burden<br>Mean oxygen saturation	Sleep fragmentation<br>Total sleep time<br>AHI total<br>Hypoxic burden<br>Mean oxygen saturation

The top-5 features are in order of their importance.

Abbreviations: AHI: apnea hypopnea index, XGB: XGBoost, N3: non-rapid eye movement sleep stage 3, NREM: non-rapid eye movement sleep stage, REM: rapid eye movement sleep stage

Supplementary Table 6: Comorbidities used as covariates for each disease in logistic and cox regression analyses

See attached file: disease_comorbidities.csv

Supplementary Table 7: Demographic and clinical characteristics of patients from different risk groups

	Risk group – No. (%) or Mean (Std)					
**Characteristic**	RG1 **(n=3357)**	RG2 **(n=1877)**	RG3 **(n=2867)**	RG4 **(n=1144)**	RG5 **(n=363)**	**P-value**
**Sociodemographics**						
**Age (years)**	50.8 (14.9)[4]	44.0 (15.0)[4]	49.7 (15.8)[4]	58.6 (16.1)[3]	58.6 (16.8)[3]	**<.001**
**Caucasian**	1581 (47.1%)	765 (40.8%)	1231 (42.9%)	575 (50.3%)	159 (43.8%)	
**African-American**	1140 (34.0%)	697 (37.1%)	952 (33.2%)	395 (34.5%)	154 (42.4%)	
**Asian**	162 (4.8%)	104 (5.5%)	170 (5.9%)	40 (3.5%)	16 (4.4%)	
**Multiracial**	429 (12.8%)	276 (14.7%)	469 (16.4%)	119 (10.4%)	32 (8.8%)	
**BMI (kg/m²)**	33.9 (8.2)[1]	32.7 (9.1)[4]	33.8 (8.7)[1]	34.4 (9.6)[1]	35.4 (10.5)[1]	**<.001**
**Male**	1766 (52.6%)	705 (37.6%)	1397 (48.7%)	696 (60.8%)	241 (66.4%)	
**Height (cm)**	170.7 (10.6)[2]	168.7 (10.5)[4]	169.9 (10.9)[4]	171.0 (11.0)[2]	171.7 (11.1)[2]	**<.001**
**Weight (kg)**	98.8 (24.9)[2]	92.9 (26.0)[4]	97.3 (25.9)[3]	100.8 (29.9)[2]	104.5 (32.3)[3]	**<.001**
**Neck Circum. (cm)**	40.1 (4.6)[3]	38.4 (4.4)[4]	39.9 (4.7)[3]	41.2 (5.4)[3]	42.1 (5.7)[3]	**<.001**
**Cardiovascular Risk Factors**						
**Diabetes Type 1**	89 (2.7%)[2]	48 (2.6%)[2]	96 (3.3%)[2]	61 (5.3%)[3]	26 (7.2%)[3]	**<.001**
**Diabetes Type 2**	1099 (32.7%)[4]	463 (24.7%)[4]	1080 (37.7%)[2]	478 (41.8%)[2]	162 (44.6%)[2]	**<.001**
**Hypertension**	2008 (59.8%)[3]	892 (47.5%)[4]	1712 (59.7%)[3]	865 (75.6%)[3]	281 (77.4%)[3]	**<.001**
**Hyperlipidemia**	1796 (53.5%)[2]	710 (37.8%)[4]	1500 (52.3%)[2]	746 (65.2%)[3]	216 (59.5%)[1]	**<.001**
**Obesity**	1490 (44.4%)[4]	681 (36.3%)[4]	1592 (55.5%)[2]	592 (51.7%)[2]	197 (54.3%)[2]	**<.001**
**Cardiovascular Disease**						
**Heart Failure**	250 (7.4%)[3]	122 (6.5%)[3]	297 (10.4%)[4]	210 (18.4%)[3]	79 (21.8%)[3]	**<.001**
**Myocardial Infarction**	142 (4.2%)[3]	59 (3.1%)[3]	190 (6.6%)[4]	111 (9.7%)[3]	44 (12.1%)[3]	**<.001**
**Atrial Fibrillation**	249 (7.4%)[3]	90 (4.8%)[4]	264 (9.2%)[3]	175 (15.3%)[3]	65 (17.9%)[3]	**<.001**
**Stroke**	319 (9.5%)[3]	137 (7.3%)[3]	395 (13.8%)[3]	206 (18.0%)[3]	70 (19.3%)[2]	**<.001**
**Ischemic Stroke**	229 (6.8%)[3]	98 (5.2%)[3]	280 (9.8%)[4]	152 (13.3%)[3]	54 (14.9%)[3]	**<.001**

**Coronary Artery Disease**	450 (13.4%)[3]	175 (9.3%)[4]	421 (14.7%)[3]	301 (26.3%)[3]	95 (26.2%)[3]	**<.001**
**Ischemic Heart Disease**	45 (1.3%)[3]	12 (0.6%)[3]	68 (2.4%)[3]	38 (3.3%)[2]	18 (5.0%)[3]	**<.001**
**Acute Coronary Disease**	2 (0.1%)	2 (0.1%)	4 (0.1%)	3 (0.3%)	0 (0.0%)	0.503
**MACE**	784 (23.4%)[4]	333 (17.7%)[4]	789 (27.5%)[4]	499 (43.6%)[3]	152 (41.9%)[3]	**<.001**
**Neurological Disorders**						
**Migraine**	486 (14.5%)[2]	369 (19.7%)[3]	565 (19.7%)[3]	131 (11.5%)[2]	39 (10.7%)[2]	**<.001**
**Alzheimer's Disease**	8 (0.2%)[1]	4 (0.2%)[1]	9 (0.3%)[1]	19 (1.7%)[3]	4 (1.1%)	**<.001**
**Mood Disorders**	1526 (45.5%)[3]	956 (50.9%)[2]	1624 (56.6%)[2]	610 (53.3%)[1]	185 (51.0%)	**<.001**
**Cognitive Impairment**	475 (14.1%)[3]	284 (15.1%)[2]	626 (21.8%)[2]	259 (22.6%)[2]	76 (20.9%)[1]	**<.001**
**Epilepsy**	172 (5.1%)[1]	129 (6.9%)	242 (8.4%)[1]	84 (7.3%)	24 (6.6%)	**<.001**
**Other Medical History**						
**COPD**	375 (11.2%)[2]	178 (9.5%)[3]	360 (12.6%)[2]	218 (19.1%)[3]	65 (17.9%)[2]	**<.001**
**Chronic Pain**	883 (26.3%)[3]	477 (25.4%)[3]	1284 (44.8%)[4]	404 (35.3%)[3]	121 (33.3%)[3]	**<.001**
**GERD**	1264 (37.7%)[2]	646 (34.4%)[2]	1256 (43.8%)[2]	486 (42.5%)[2]	141 (38.8%)	**<.001**
**CABG**	69 (2.1%)[2]	30 (1.6%)[2]	74 (2.6%)[2]	63 (5.5%)[3]	24 (6.6%)[3]	**<.001**
**Chronic Insomnia**	315 (9.4%)[3]	184 (9.8%)[3]	547 (19.1%)[2]	195 (17.0%)[2]	64 (17.6%)[2]	**<.001**
**PSG results**						
**AHI Total**	12.4 (9.9)[4]	5.4 (6.4)[4]	11.2 (12.3)[4]	22.7 (24.4)[4]	37.3 (39.0)[4]	**<.001**
**AHI Off-supine**	8.3 (9.5)[3]	3.5 (7.0)[4]	7.9 (11.2)[3]	18.1 (23.6)[4]	35.6 (40.4)[4]	**<.001**
**AHI Supine**	21.3 (23.0)[4]	10.2 (16.5)[4]	18.3 (22.2)[4]	31.6 (32.6)[4]	41.4 (44.4)[4]	**<.001**
**Arousal Index**	23.8 (12.2)[4]	19.3 (11.1)[4]	22.7 (13.2)[4]	41.5 (24.4)[4]	60.8 (42.0)[4]	**<.001**
**CAI**	0.3 (1.2)[4]	0.1 (0.4)[4]	0.5 (1.9)[4]	1.4 (5.6)[3]	3.3 (12.2)[3]	**<.001**
**Central Apneas**	1.7 (6.2)[4]	0.9 (4.8)[4]	2.5 (9.9)[3]	5.1 (23.9)[3]	5.5 (24.3)[2]	**<.001**
**Hypopneas**	57.8 (48.9)[3]	27.2 (32.5)[3]	48.8 (56.7)[3]	57.8 (76.7)[3]	37.8 (84.4)[2]	**<.001**
**Maximum $EtCO_2$**	48.7 (10.3)[1]	49.4 (20.0)[1]	49.0 (9.0)[2]	48.1 (6.5)[2]	46.4 (8.3)[4]	**<.001**
**Mean O_2 Saturation**	94.0 (2.1)[3]	95.4 (1.6)[4]	94.2 (2.2)[3]	93.6 (2.5)[3]	93.4 (3.0)[3]	**<.001**
**Min O_2 Saturation**	84.9 (6.2)[2]	89.0 (4.9)[4]	85.0 (7.6)[2]	84.1 (7.6)[3]	83.8 (10.7)[1]	**<.001**
**NREM AHI**	9.5 (9.6)[3]	3.7 (5.8)[4]	9.2 (11.9)[3]	20.9 (24.6)[4]	37.1 (39.7)[4]	**<.001**
**Obstructive Apneas**	7.7 (15.9)[3]	2.2 (6.6)[4]	7.4 (17.2)[3]	16.8 (38.5)[3]	21.2 (59.9)[3]	**<.001**
**REM AHI**	24.9 (21.2)[4]	11.9 (14.5)[4]	22.3 (21.8)[4]	30.9 (29.0)[3]	36.3 (36.8)[3]	**<.001**
**Sleep Stage N1 (%)**	8.1 (5.6)[4]	7.0 (5.6)[4]	8.6 (6.2)[4]	16.7 (12.6)[4]	33.7 (27.1)[4]	**<.001**
**Sleep Stage N2 (%)**	68.3 (10.3)[4]	66.5 (12.1)[4]	71.1 (9.9)[3]	70.5 (14.3)[3]	57.5 (26.8)[4]	**<.001**
**Sleep Stage N3 (%)**	6.8 (8.1)[4]	10.1 (10.4)[4]	4.7 (7.3)[4]	3.9 (7.9)[3]	2.5 (8.1)[3]	**<.001**
**Sleep Stage REM (%)**	17.4 (7.5)[3]	17.1 (8.1)[3]	15.9 (7.6)[4]	9.4 (9.0)[4]	3.9 (8.1)[4]	**<.001**
**Sleep Time SpO_2 Under 90% (%)**	7.1 (19.8)[4]	2.0 (10.2)[4]	11.7 (33.6)[2]	14.9 (33.8)[2]	14.4 (33.2)[2]	**<.001**
**Snoring**	3076 (91.8%)[2]	1588 (84.9%)[4]	2563 (90.1%)[2]	1013 (89.3%)[2]	276 (76.5%)[4]	**<.001**
**Total Sleep Time (min)**	328.7 (61.1)[4]	342.6 (71.6)[4]	321.4 (59.9)[4]	201.3 (73.9)[4]	98.4 (89.0)[4]	**<.001**
**Total NREM time (min)**	270.3 (48.8)[3]	282.6 (57.2)[4]	269.4 (50.2)[3]	181.7 (67.5)[4]	91.8 (78.6)[4]	**<.001**
**Total REM time (min)**	58.6 (29.8)[3]	60.2 (34.1)[3]	52.2 (28.5)[4]	19.7 (21.2)[4]	6.9 (16.5)[4]	**<.001**
**Alternative metrics**						
**Sleep Fragmentation**	0.2 (0.1)[3]	0.2 (0.1)[4]	0.2 (0.1)[3]	0.4 (0.1)[4]	0.7 (0.2)[4]	**<.001**
**Hypoxic Burden**	24.8 (22.1)[4]	8.5 (10.1)[4]	27.5 (32.6)[4]	55.9 (69.2)[4]	128.7 (164.6)[4]	**<.001**

**Lung 2 finger time**	20.5 (6.3)^{2}	20.0 (7.7)^{2}	20.2 (7.0)^{2}	22.5 (8.2)^{3}	23.8 (10.4)^{3}	**<.001**
**Delta HR**	12.8 (5.6)	12.8 (5.9)	13.1 (5.9)	12.6 (7.5)	13.8 (8.6)	0.062
**Other**						
**ESS**	8.7 (5.3)^{3}	9.4 (5.6)^{4}	8.7 (5.3)^{3}	7.7 (5.2)^{3}	7.9 (5.4)^{3}	**<.001**
**Average Sleep⁺ (hours)**	6.2 (2.1)	6.4 (1.9)	6.5 (2.0)	6.3 (2.4)	5.9 (2.2)	0.189

Abbreviations: AHI: Apnea Hypopnea Index; BMI: Body Mass Index; CABG: Coronary Artery Bypass Grafting; CAI: Central Apnea Index; COPD: Chronic Obstructive Pulmonary Disease; ESS: Epworth Sleepiness Scale; $EtCO_2$: End-Tidal Carbon Dioxide; GERD: Gastroesophageal Reflux Disease; HR: Heart Rate; MACE: Major Adverse Cardiovascular Events; NREM: Non-Rapid Eye Movement; O_2: Oxygen; REM: Rapid Eye Movement

Superscript numbers indicate the number of groups from which the value is significantly different. ⁺Average sleep is self-reported and was available for only 1,822 samples.

Supplementary Table 8: Definitions of sleep variables

Variable	Definition
**REM**	Rapid eye movement sleep stage
**NREM**	Non-rapid eye movement sleep stages
**N1**	Non-rapid eye movement sleep stage 1
**N2**	Non-rapid eye movement sleep stage 2
**N3**	Non-rapid eye movement sleep stage 3
**AHI, total**	Apnea hypopnea index; Respiratory events per hour of sleep
**AHI, supine**	Respiratory events per hour of supine sleep
**AHI, off-supine**	Respiratory events per hour of off-supine sleep
**REM AHI**	Respiratory events per hour of REM sleep
**NREM AHI**	Respiratory events per hour of NREM sleep
**Obstructive apnea**	≥10 seconds of ≥90% airflow reduction from pre-event baseline with continued respiratory effort
**Central apnea**	≥10 seconds of ≥90% airflow reduction from pre-event baseline without respiratory effort
**Mixed apnea**	≥10 seconds of ≥90% airflow reduction from pre-event baseline without respiratory effort for a portion of time and with respiratory effort for a portion of time
**Hypopnea**	≥10 seconds of ≥30% reduction in the nasal transducer amplitude from pre-event baseline associated with either a 4% oxygen desaturation or a 3% oxygen desaturation or arousal*
**Central apnea index**	The number of central apneas per hour of sleep
**Arousal index**	Number of arousals per hour of sleep
**Epworth Sleepiness Scale**	Self-reported questionnaire that measures daytime sleepiness based on the likelihood of dozing off in various daily situations
**Total sleep time**	Total sleep time; expressed in minutes
**Sleep fragmentation**	The normalized power in the "fast" frequency range of the hypnogram's power spectral density (PSD), specifically transitions occurring faster than 10 minutes
**Sleep apnea-specific hypoxic burden**	The area under the curve of desaturations temporally related to respiratory events
**Delta Heart Rate**	Heart rate response; change in heart rate following respiratory events
**Lung 2 finger time**	Time between the end of a respiratory event and the lowest point of oxygen desaturation

*Depending on insurer, as this is a clinical cohort

Supplementary Table 9: Logistic Regression (Odds Ratios, 95% Confidence Intervals) of Comorbidities among Patients from Different Risk Groups

	Risk group			
**Disease**	RG**2 (n=1877)**	RG**3 (n=2867)**	RG**4 (n=1144)**	RG**5 (n=363)**
**Cardiovascular Risk Factors**				
**Hypertension**	1.02 (0.86-1.19)	1.26 (1.10-1.45)$^{(***)}$	1.22 (1.00-1.50)	1.36 (0.95-1.95)
**Diabetes Type 2**	0.82 (0.70-0.96)$^{(*)}$	1.36 (1.20-1.53)$^{(***)}$	1.19 (1.01-1.40)$^{(*)}$	1.33 (1.03-1.73)$^{(*)}$
**Hyperlipidemia**	0.77 (0.65-0.90)$^{(**)}$	1.10 (0.96-1.25)	1.00 (0.83-1.21)	0.72 (0.53-0.98)$^{(*)}$
**Obesity**	0.81 (0.67-0.98)$^{(*)}$	2.49 (2.13-2.91)$^{(***)}$	1.68 (1.36-2.06)$^{(***)}$	1.59 (1.13-2.23)$^{(**)}$
**Cardiovascular Disease**				
**MACE**	1.04 (0.86-1.26)	1.53 (1.33-1.77)$^{(***)}$	1.64 (1.36-1.96)$^{(***)}$	1.41 (1.05-1.89)$^{(*)}$
**Heart Failure**	1.10 (0.83-1.46)	1.81 (1.47-2.23)$^{(***)}$	1.86 (1.47-2.36)$^{(***)}$	2.11 (1.49-2.98)$^{(***)}$
**Myocardial Infarction**	1.02 (0.71-1.48)	2.22 (1.71-2.89)$^{(***)}$	1.46 (1.07-1.99)$^{(*)}$	1.85 (1.19-2.87)$^{(**)}$
**Ischemic Heart Disease**	0.60 (0.28-1.25)	2.21 (1.44-3.38)$^{(***)}$	1.64 (1.01-2.68)$^{(*)}$	2.00 (1.04-3.85)$^{(*)}$
**Coronary Artery Disease**	0.99 (0.79-1.25)	1.38 (1.16-1.64)$^{(***)}$	1.38 (1.12-1.70)$^{(**)}$	1.16 (0.84-1.60)
**Atrial Fibrillation**	0.89 (0.66-1.20)	1.47 (1.19-1.82)$^{(***)}$	1.54 (1.21-1.96)$^{(***)}$	1.68 (1.18-2.41)$^{(**)}$
**Stroke**	1.03 (0.80-1.32)	1.76 (1.47-2.12)$^{(***)}$	1.44 (1.16-1.81)$^{(**)}$	1.52 (1.08-2.13)$^{(*)}$
**Ischemic Stroke**	1.06 (0.79-1.41)	1.74 (1.41-2.16)$^{(***)}$	1.45 (1.13-1.87)$^{(**)}$	1.53 (1.04-2.23)$^{(*)}$
**Neurological Disorders**				
**Mood Disorders**	1.00 (0.87-1.16)	1.59 (1.40-1.80)$^{(***)}$	1.58 (1.34-1.87)$^{(***)}$	1.67 (1.29-2.18)$^{(***)}$
**Migraine**	1.03 (0.86-1.24)	1.34 (1.15-1.56)$^{(***)}$	0.96 (0.76-1.22)	0.96 (0.64-1.42)
**Cognitive Impairment**	1.15 (0.94-1.39)	1.74 (1.50-2.03)$^{(***)}$	1.50 (1.24-1.83)$^{(***)}$	1.45 (1.06-1.97)$^{(*)}$
**Epilepsy**	1.09 (0.83-1.44)	1.58 (1.27-1.98)$^{(***)}$	1.62 (1.21-2.18)$^{(**)}$	1.61 (1.00-2.58)$^{(*)}$
**Other**				
**GERD**	0.98 (0.85-1.14)	1.33 (1.18-1.49)$^{(***)}$	1.15 (0.98-1.35)	1.02 (0.79-1.32)
**Acute Pain**	0.58 (0.41-0.82)$^{(**)}$	2.71 (2.18-3.38)$^{(***)}$	1.57 (1.15-2.14)$^{(**)}$	1.25 (0.73-2.15)
**Chronic Pain**	0.86 (0.74-1.01)	2.45 (2.16-2.77)$^{(***)}$	1.61 (1.37-1.90)$^{(***)}$	1.45 (1.11-1.89)$^{(**)}$

Disease abbreviations: MACE: Major Adverse Cardiovascular Events; GERD: Gastroesophageal Reflux Disease

Significance levels: *p<0.05; **p<0.01; ***p<0.001

Supplementary Table 10: Logistic regression results for each disease at baseline

See attached file: logistic_summary_results.xlsx

Supplementary Table 11: Disease vocabulary used to parse the EMR data

See attached file: disease_vocabulary.xlsx
